# Active shape programming drives *Drosophila* wing disc eversion

**DOI:** 10.1126/sciadv.adp0860

**Published:** 2024-08-09

**Authors:** Jana F. Fuhrmann, Abhijeet Krishna, Joris Paijmans, Charlie Duclut, Greta Cwikla, Suzanne Eaton, Marko Popović, Frank Jülicher, Carl D. Modes, Natalie A. Dye

**Affiliations:** ^1^ Max-Planck Institute for Molecular Cell Biology and Genetics, MPI-CBG, Pfotenhauerstrasse 108, 01307 Dresden, Germany.; ^2^ Cluster of Excellence Physics of Life, Technische Universität Dresden, Arnoldstrasse 18, 01307 Dresden, Germany.; ^3^ Center for Systems Biology, Pfotenhauerstrasse 108, 01307 Dresden, Germany.; ^4^ Max-Planck Institute for Physics of Complex Systems, MPI-PKS, Nöthnitzer Str. 38, 01187 Dresden, Germany.; ^5^ Laboratoire Physico-Chimie Curie, CNRS UMR 168, Institut Curie, Université PSL, Sorbonne Université, 75005 Paris, France.; ^6^ Biotechnologisches Zentrum, Technische Universität Dresden, Tatzberg 47-49, 01307 Dresden, Germany.; ^7^ Mildred Scheel Nachwuchszentrum P2, Medical Faculty, Technische Universität Dresden, Dresden, Germany.

## Abstract

How complex 3D tissue shape emerges during animal development remains an important open question in biology and biophysics. Here, we discover a mechanism for 3D epithelial shape change based on active, in-plane cellular events that is analogous to inanimate “shape programmable” materials, which undergo blueprinted 3D shape transformations from in-plane gradients of spontaneous strains. We study eversion of the *Drosophila* wing disc pouch, when the epithelium transforms from a dome into a curved fold, quantifying 3D tissue shape changes and mapping spatial patterns of cellular behaviors on the evolving geometry using cellular topology. Using a physical model inspired by shape programming, we find that active cell rearrangements are the major contributor to pouch eversion and validate this conclusion using a knockdown of MyoVI, which reduces rearrangements and disrupts morphogenesis. This work shows that shape programming is a mechanism for animal tissue morphogenesis and suggests that patterns in nature could present design strategies for shape-programmable materials.

## Teaser

In-plane cellular dynamics drive 3D tissue morphogenesis of the everting *Drosophila* wing disc epithelium.

## INTRODUCTION

Epithelial tissues are sheets of tightly connected cells with apical-basal polarity that form the basic architecture of many animal organs. Deformations of animal epithelia in 3D can be mediated by external forces, either from neighboring tissue that induces buckling instabilities [e.g., (*1*–*3*)] or extracellular matrix that confines [e.g., (*4*)] or expands [e.g., (*5*)]. Alternatively, local differences in mechanics at the apical and basal sides of the deforming epithelia itself can drive out-of-plane tissue shape changes {e.g., ventral furrow invagination in the *Drosophila* embryo [reviewed in (*6*)] and fold formation in *Drosophila* imaginal discs (*7*)}.

Here, we describe a mechanism for generating complex three-dimensional (3D) tissue shape involving tissue-scale patterning of in-plane deformations, analogous to the shape transformations of certain inanimate shape-programmable materials. These shape-programmable materials, like hydrogels and nematic elastomers, experience spontaneous strains, where the natural internal lengths change in response to stimuli in a desired way (*8*, *9*). Globally patterned spontaneous strains can create a geometric incompatibility with the original shape, triggering specific, desired 3D deformations, such as the formation of a cone from a flat sheet (*8*, *10*, *11*). Ideas from shape programmability have already proved insightful to the understanding of differential growth-mediated plant morphogenesis (*12*, *13*). However, animal epithelia are more dynamic, changing cell shape and size, as well as rearranging tissue topology. Each of these behaviors could actively create in-plane spontaneous strain or be a passive response to stress.

**Fig. 1.**
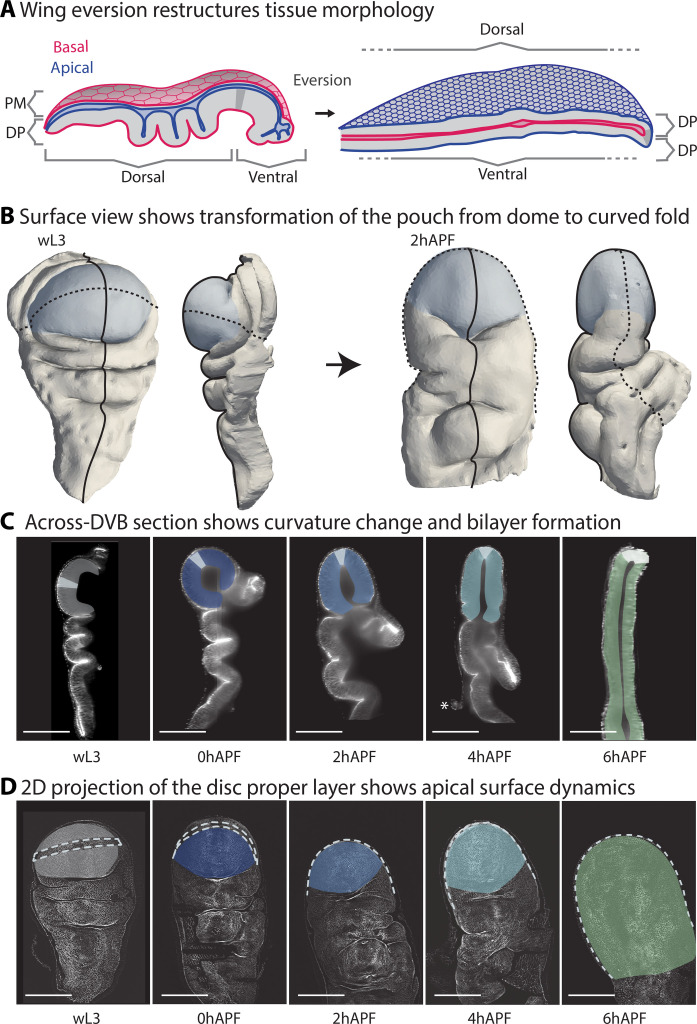
The wing pouch undergoes anisotropic curvature changes during eversion. (**A**) Schematic cross sections along the long axis of the wing disc before and after eversion. DP, disc proper; PM, peripodial membrane; blue, apical surfaces; red, basal surfaces. (**B**) Example of a 3D segmentation of the DP in a head-on and side view (from anterior) before eversion (left, wL3) and after bilayer formation (right, 2hAPF). Blue, pouch; solid line, across-DVB; dashed line, along-DVB. (**C** and **D**) Representative images of wing discs during eversion. Wing discs are labeled with E-cadherin–GFP. The pouch region is highlighted, colored by time. (C) Projection view showing the dorsal side for early pupal stages and dorsal (down), ventral (up), and DVB (dashed line) for wL3. (D) Across-DVB cross section (white: DVB). Asterisk shows the rupture point of the PM, which gets removed around 4hAPF. Minimum five wing discs were analyzed for each time point. Scale bars, 100 μm.

To test whether shape programming could be a mechanism for animal morphogenesis, we quantify tissue shape changes and cell behaviors in the *Drosophila* wing disc during a 3D morphogenetic process called eversion (Fig. 1A). Through eversion, the wing disc proper, an epithelial monolayer, undergoes a shape deformation in which the future dorsal and ventral surfaces of the wing blade appose to form a bilayer and escape the overlying squamous epithelium called the peripodial membrane. After eversion, the wing disc begins to resemble the final shape of the adult wing. This process is triggered by a peak in circulating levels of the hormone 20-hydroxyecdysone, analogous to an activator in shape programming. This complex tissue shape change is independent of forces external to the wing disc, as demonstrated by its ability to occur in explant culture (*14*). The shape changes of the disc proper also cannot be fully explained by removal of the peripodial membrane or extracellular matrix and appear to be self-sufficient, involving active cellular processes (*15*–*19*).

It has long been postulated that the eversion of wing (and leg) discs is achieved by in-plane cell behaviors that are organized by previously established cell morphology patterns (*20*–*23*). Here, we test this hypothesis by systematic quantification and genetic perturbation of cell behaviors during eversion and demonstrate how cell behaviors contribute to tissue shaping using a physical model analogous to shape programming.

## RESULTS

### The wing pouch undergoes anisotropic curvature changes during eversion

We first sought to quantify the tissue shape changes happening during wing disc eversion. To this end, we explanted wing discs at fixed time intervals, from late larval stage [wandering third larval instar (wL3)] to 6 hours after puparium formation (hAPF). We imaged the wing discs using multiangle light sheet microscopy and then reconstructed and analyzed the 3D image stack (the “Image acquisition and processing” section). In this way, we capture the complex 3D shape changes happening throughout the wing disc during eversion (Fig. 1B and movie S1).

The most marked tissue shape changes can be seen in a central cross section along the axis perpendicular to the dorsal ventral boundary (DVB), referred to as “across-DVB” (Fig. 1C and fig. S1, D, F, and G). We observe three main morphogenetic changes: The peripodial membrane is removed around 4hAPF, the deeply folded regions unfold, and the pouch undergoes a transition from a monolayer dome to a flat bilayer with a sharply folded interface. In the perpendicular plane, taken through DVB in the pouch (referred to as “along-DVB”), the tissue does not change as considerably, preserving curvature in this direction (Fig. 1D and fig. S1, E to G).

We focus hereafter on the pouch region, as it undergoes the most complex shape change: starting as an almost radially symmetric dome and ending up in a curved fold shape, with curvature increasing strongly in one axis (across-DVB) but not as much in the other (along-DVB) (fig. S1, F and G). To test the hypothesis that in-plane cellular behaviors lead to 3D tissue shape change, we first build a shape programmability model that relates cellular behaviors to spontaneous strain. We then measure patterns of cellular behaviors in the wing pouch during eversion and use our model to test how they affect tissue shape change.

### Programmable spring network as a model for epithelial morphogenesis

**Fig. 2.**
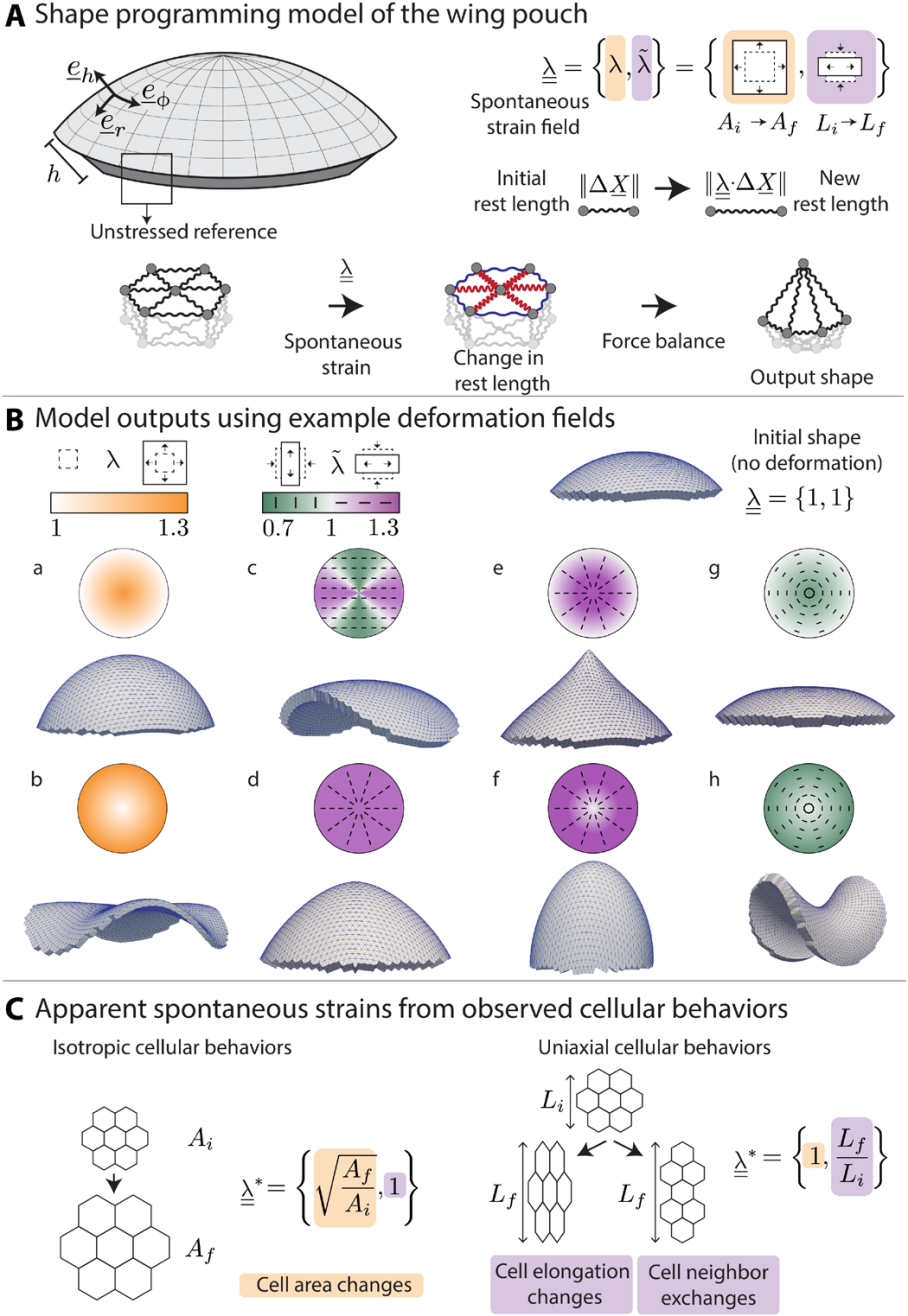
The programmable spring network relates cell behaviors to spontaneous strain to model epithelial morphogenesis. (**A**) A thick spherical cap as a model for an epithelial tissue; radial coordinate *r* and basis vectors ${\underline{e}}_{r},{\underline{e}}_{\phi },$ and ${\underline{e}}_{h}$. The thickness (*h*) is constant everywhere. The model tissue is an elastic medium implemented as a spring network with an initially stress-free state. We change the rest lengths of the springs by imposing a spontaneous strain field $\underline{\underline{\lambda }}$ and allow subsequent relaxation leading to a new 3D output shape. The spontaneous strain field $\underline{\underline{\lambda }}$ consists of an isotropic component λ and an anisotropic component $\tilde{\lambda }$ . These components cause changes in local area (*A_i_* to *A_f_*) or local shape (*L_i_* to *L_f_*), respectively. (**B**) Model realizations with example spontaneous strain patterns. The input pattern is displayed above, with the magnitude of spontaneous strain encoded by color and the orientation of anisotropic extension displayed in the bars. The rest lengths of the springs (mesh edges) are changed according to the input spontaneous strain pattern. The output shape is displayed below. In (B, a and b), we vary the isotropic contribution λ and keep $\tilde{\lambda }=1$ , while in (B, c to h), we vary $\tilde{\lambda }$ and keep λ = 1. (**C**) Schematics showing the calculation of observed strains from cellular behaviors. Left: For a patch of cells going from area of *A_i_* to *A_f_*, we extract λ*. Right: A patch of cells undergoing anisotropic deformation due to cell elongation changes or neighbor exchanges causes the length scale in one direction to change from *L_i_* to *L_f_*. From this change, ${\tilde{\lambda }}^{*}$ is extracted, while λ* = 1, as there is no isotropic contribution.

We developed a coarse-grained model of tissue shape changes, leveraging an analogy between tissue remodeling by internal processes and spontaneous strain-driven shape programming of nematic elastomers (*24*–*26*). We use a double layer of interconnected programmable springs representing the apical surface geometry and the material properties of an epithelial sheet, including a bending rigidity introduced by the thickness of the double layer (Fig. 2A and the “Mechanics of the programmable spring lattice” and the “Tuning thickness” sections). As an initial configuration, we use a stress-free spherical cap and then assign new rest lengths to the springs. In a continuum limit, this corresponds to introducing a spontaneous strain field $\underline{\underline{\lambda }}\;\left(\underline{X}\right)$, which depends on the spatial coordinates $\underline{X}$ (the “Spontaneous strain tensor” section). To simplify notation, we write $\underline{\underline{\lambda }}$ for $\underline{\underline{\lambda }}\;\left(\underline{X}\right)$ hereafter. To generate a final output shape, we quasi-statically relax the spring network (the “Mechanics of the programmable spring lattice” and the “Spontaneous strain tensor” sections). As with conventional elastic strain tensors, $\underline{\underline{\lambda }}$ can be decomposed into isotropic (λ) and anisotropic ($\tilde{\lambda }$) modes.

We first demonstrate how simple choices of spontaneous strain patterns induce a shape change in our model. A simple gradient of λ, for example, causes the spherical cap to balloon in the center or generate wrinkles at the periphery (Fig. 2B, a and b). Changing the directions and gradients of $\tilde{\lambda }$ leads to elongation of the cap, increase in the curvature at the tip, or even flattening of the curvature in the center, eventually leading to a saddle shape (Fig. 2B, c to h).

Next, we propose that cell behaviors can give rise to a spontaneous strain field, thereby shape programming the wing disc pouch and driving 3D shape changes during eversion. The strains measured from observed cell behaviors during eversion (referred to as observed strains, ${\underline{\underline{\lambda }}}^{*}$ ) can be used to infer spontaneous strains. By using coarse-grained spontaneous strains, the topology of the spring network remains unchanged (*27*). For the isotropic component of observed strain, we focus on cell area changes ( $\lambda_{A}^{*}$ ) (Fig. 2C). The anisotropic components of observed strain capture contributions stemming from both changes in cell elongation (${\tilde{\lambda }}_{Q}^{*}$) as well as from cell rearrangements (${\tilde{\lambda }}_{R}^{*}$), which can include cell divisions, cell extrusions, and T1 transitions (Fig. 2C). Our model can therefore relate cell behaviors to spontaneous strains to understand resulting tissue deformations. We now investigate these quantities in the everting wing disc.

### Topological tracking reveals spatial patterns of cell dynamics

**Fig. 3.**
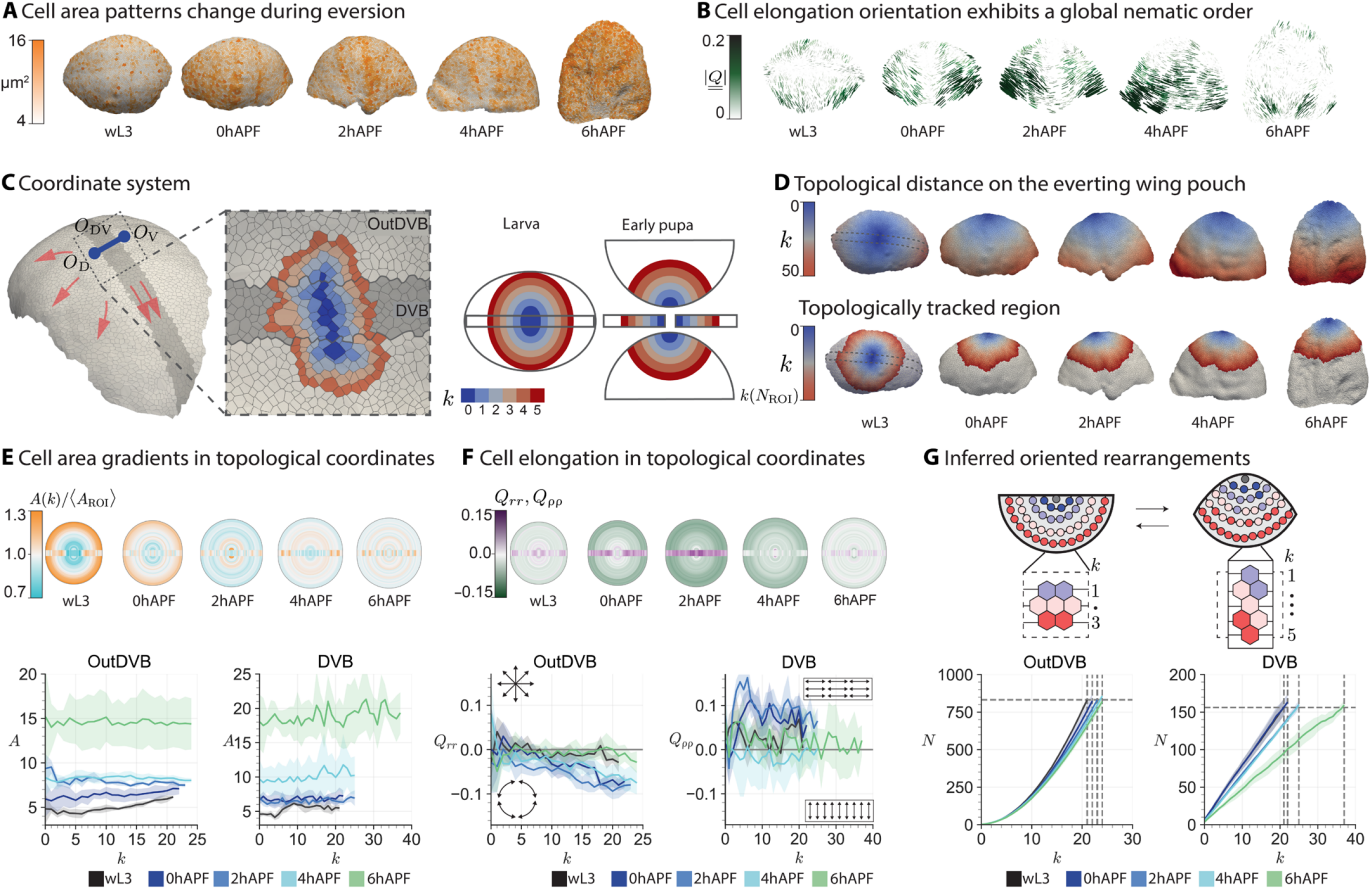
Topological tracking reveals spatial patterns of change in cell size, cell elongation, and cell rearrangements. (**A**, **B**, and **D**) Cell measurements on the surface of example wing discs. At wL3, outDVB and DVB are visible; for 0hAPF to 6hAPF, dorsal is shown. (A) Apical cell area. (B) Cell elongation averaged over patches of 350 μm^2^. Bars, orientation of $\underline{\underline{Q}}$ (2D projected); color, magnitude of ($|\underline{\underline{Q}}|$). (**C**) Segmentation of a wL3 pouch. Blue, origins (outDVB: *O*_D_ and *O*_V_ and DVB: *O*_DV_) for topological coordinates *k*; orange arrows, direction of spatial coordinates emanating from these origins. Inset: Center region colored by *k*. The topological coordinate system is defined in one view for larval stages and in four separate imaging angles for early pupal stages (schematic, right). (D) Cells colored by *k* (top row) and by *k* limited to the topologically tracked region (maximum *k* is denoted *k*[*N*_ROI_]) (bottom row). (**E** and **F**) Cell area (E) or elongation (F) averaged over *k*. Geometric representations (half-circles: outDVB, rectangle: DVB, radius: *k*). (E) Geometry: Cell area normalized per time point and region [*A*(*k*)/〈*A*_ROI_〉]. Bottom: Cell area (*A*) over *k*. (F) Radial component of cell elongation (**Q*_*rr*_*) for outDVB cells and cell elongation along the DVB (*Q*_ρρ_) for DVB cells. **Q*_*rr*_* and *Q*_ρρ_ are calculated as a function of *k*. In the top, magnitudes are represented by color. (**G**) Schematic shows rearrangement estimate. Circles represent outDVB cells, colored by *k* at the initial time point. Radial rearrangements lead to a decrease, tangential to an increase in the number of cells per *k*. Plots show the cumulative number of cells *N* contained within *k*. Horizontal line, *N*_ROI_ for wL3; vertical lines, corresponding *k*(*N*_ROI_) for each stage. In (E) to (G), solid lines indicate mean, and ribbons show 95% confidence of the mean.

To examine cell behaviors, we first segmented apical cell junctions and plotted average cell area and cell elongation in space (fig. S2 and Fig. 3, A and B). From larval stages, we know that cell morphology and behaviors in the pouch are organized radially in the region outside of the dorsal ventral boundary (outDVB) and parallel to the boundary in the region closest to the DVB (*28*–*31*). During eversion, we observe that cell shapes and sizes are patterned similarly. In early stages, cell area follows a radial gradient that disappears by the end of eversion (4hAPF) (Fig. 3A and the “3D cellular network” section). Cell elongation exhibits a global nematic order through 4hAPF before disordering at 6hAPF (Fig. 3B and the “3D cellular network” section).

To compare spatial patterns of cell behaviors over eversion time and across experiments, we define a coordinate system on the evolving 3D geometry. To this end, we use the cellular network topology to define a distance measure on the tissue surface. The topological distance between two cells is defined as the number of cells on the shortest path through the network from one cell to the other (see fig. S3A). We then use topological distance to define a coordinate system in the outDVB and DVB regions (Fig. 3C; figs. S3 and S4, A and B; and the “Topological distance coordinate system” section). The outDVB region consists of the dorsal and ventral halves, and we identify a single cell that defines the origin in each half (*O*_D_ and *O*_V_). In the DVB, we define the origin (*O*_DV_) as a line of cells transversing the DVB. The topological distance *k* to the origin defines a radial topological coordinate in each region (Fig. 3, C and D).

During eversion, tissue previously hidden in the folds becomes visible. To compare cell behaviors at different time points, we need to identify a region of tissue that remains in the field of view throughout eversion. To this end, we count *N*_ROI_, which is the total number of cells enclosed by the largest visible topological ring at wL3. The corresponding region of interest (ROI) at later time points is then defined to be centered at the origin and containing the same number of cells. Since there are very few divisions (fig. S5) (*14*, *32*, *33*) and extrusions (*34*) during early pupal eversion stages (0hAPF to 4hAPF), and because cells cannot flow across the DVB (*35*, *36*), we expect that our ROIs contain largely the same set of cells, and we refer to them as topologically tracked regions (Fig. 3D and fig. S6, A and B).

Next, we quantify patterns of cell area (*A*) and radial cell elongation tensor ($\underline{\underline{Q}}$) as a function of topological coordinate *k* throughout eversion (see the “3D cellular network” section). We find that our topological coordinate system recapitulates previously reported gradients in cell area and radial cell elongation at earlier larval stages (fig. S4, C and D). In outDVB at wL3, we observe a cell area gradient that relaxes gradually until 4hAPF (Fig. 3E). At the same time, cell elongation develops a gradient, with cells in the periphery elongating tangentially (Fig. 3F). Between 4hAPF and 6hAPF, cells markedly expand their area and tangential cell elongation completely relaxes. We do not observe gradients in cell area or cell elongation in the DVB. Instead, cell area expands globally in the DVB during eversion, while cell elongation along the DVB first increases up to 2hAPF and then decreases at 4hAPF (Fig. 3, E and F).

Using topological distance allows us to extract spatial patterns of oriented cell rearrangements from snapshots of eversion. Radially oriented rearrangements lead to a decrease in the number of cells per *k*, whereas tangentially oriented rearrangements lead to an increase. As a consequence, *k*(*N*_ROI_), which describes the required *k* covering a fixed number of cells over time, has to increase if rearrangements are radial and decrease if rearrangements are tangential. We find that *k*(*N*_ROI_) increases with time (Fig. 3G), consistent with radially oriented cell rearrangements in the outDVB and rearrangements oriented along the boundary in the DVB. To further validate our method, we performed live imaging of an everting wing disc in culture (see also movie S2). We directly observe cell intercalations and find good agreement between topological tracking and live cell tracking that is consistent with a radial bias in orientation of intercalations (fig. S7).

Together, these measured cell behaviors are a superposition of different radial patterns with the additional complexity of the DVB. Next, using our programmable spring model (Fig. 2), we ask how in-plane strains caused by these cell behaviors could drive 3D shape changes during eversion.

### Active cell rearrangements drive tissue shape changes

**Fig. 4.**
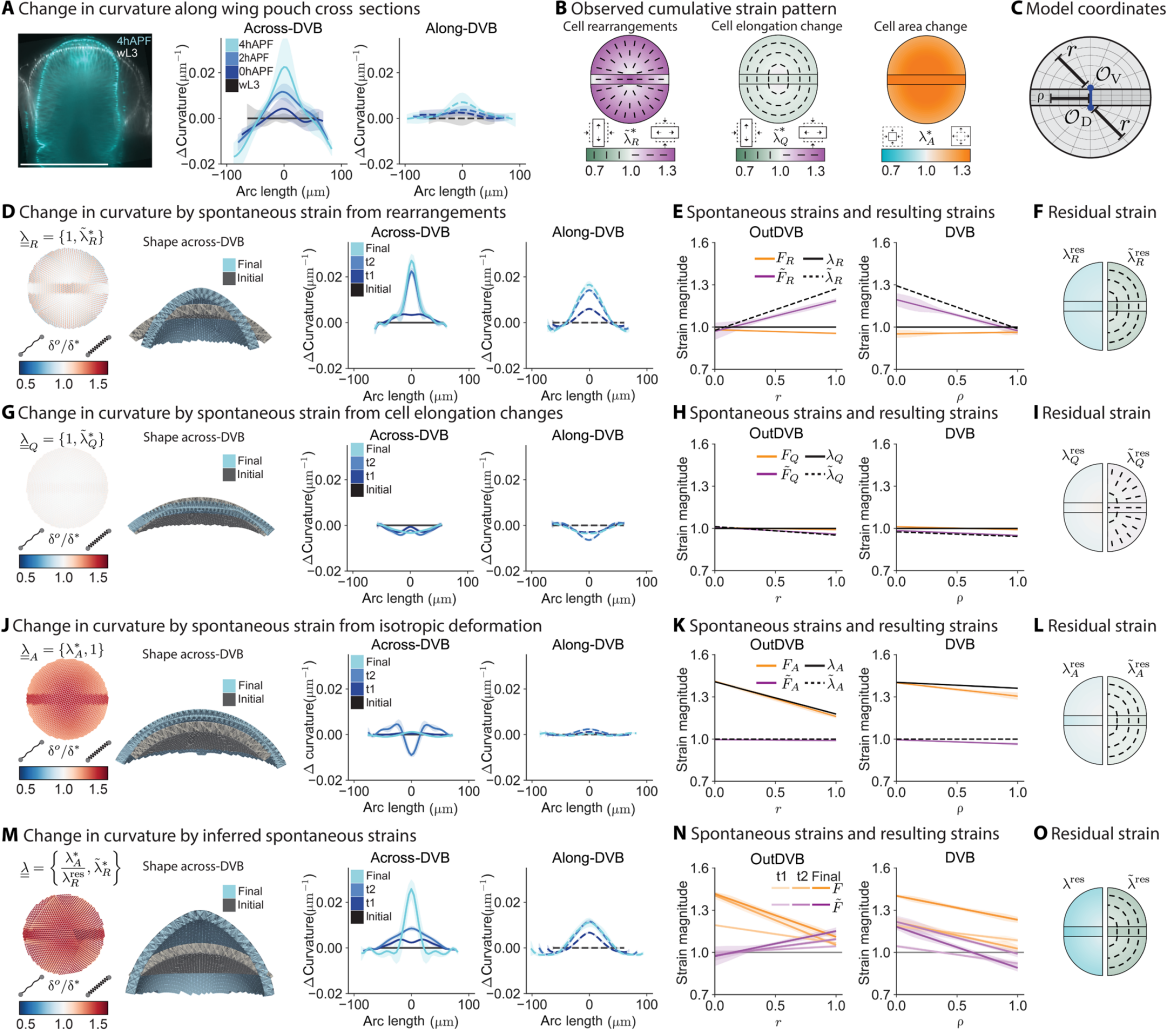
Inputting measured and inferred strains in the programmable spring model shows that active rearrangements and cell area changes drive pouch morphogenesis. (**A**) Overlay of a wL3 and 4hAPF wing pouch in across-DVB cross section (left) and plots of the average change in tissue curvature in the topologically tracked region for across-DVB (middle) and along-DVB (right) directions. (**B**) Observed strain arising from different cellular behaviors between wL3 and 4hAPF. Half circles, outDVB; rectangle, DVB. Color represents the magnitude of strain; bars visualize the orientation for ${\tilde{\lambda }}_{R}^{*}$ and ${\tilde{\lambda }}_{Q}^{*}$. (**C**) Model coordinates match the geometry of the pouch. *r* and ρ are the normalized distances from the origins. (**D**, **G**, and **J**) Observed in-plane strain from rearrangements [(D), ] ${\tilde{\lambda }}_{R}^{*}$, cell elongation changes [(G), ]${\tilde{\lambda }}_{Q}^{*}$, and cell area changes [(J),] $\lambda_{A}^{*}$ are inserted in the model as spontaneous strains. Springs are colored by new rest lengths normalized by the initial rest lengths (δ*^o^*/δ^*^). Model cross sections show the shape in the across-DVB direction for initial and final time points. Curvature change of model outcomes are plotted (right) in the across-DVB and along-DVB directions. t0 matches wL3 shape; t1, t2, and final stages are the model results from the change in strains at 0hAPF, 2hAPF, and 4hAPF. (**E**, **H**, and **K**) Input spontaneous strain for ${\tilde{\lambda }}_{R}^{*}$ (E), ${\tilde{\lambda }}_{Q}^{*}$ (H), and $\lambda_{A}^{*}$ (K) at the final eversion time point (λ, $\tilde{\lambda }$ ) and the resulting strain that is achieved after relaxation of the model, both isotropic (*F*) and anisotropic ( $\tilde{F}$ ) components. (**F**, **I**, and **L**) Residual strain that remains at the final time point. Plots are split vertically to show the isotropic component (λ) on the left and the anisotropic component ( $\tilde{\lambda }$ ) on the right. (**M** to **O**) Model output, resulting strains, and residual strains for the inferred spontaneous strains, following (D) to (L).

To be able to compare the output of the model to the 3D shape changes happening during eversion, we quantify the curvature and size dynamics of the apical surface of the wing pouch. We limit the analysis to the topologically tracked region and quantify the change in curvature from the wL3 stage along lines in the along-DVB and across-DVB directions (Fig. 4A, fig. S6, and the “Quantifying curvature of cross sections” section). We focus on the stages between wL3 and 4hAPF, during which cell shape patterns have radial symmetry (Fig. 3, A, B, E, and F). At the wL3 stage, this region is symmetric in the center and becomes slightly asymmetric in the periphery. Over eversion time, there is an overall curvature increase that is more pronounced in the across-DVB direction, peaking at the DVB, while flattening at the dorsal and ventral sides. Furthermore, the overall tissue area increases (Fig. 4A and fig. S6A).

Next, we measure the strain field ( ${\underline{\underline{\lambda }}}^{*}$ ) resulting from cell behaviors as a function of the normalized distance from the origin, *r* or ρ (Fig. 4, B and C; figs. S8 and S9; and the “Extracting the strain pattern from segmented images” section). We quantify the isotropic component of strain resulting from cell area changes ( $\lambda_{A}^{*}$ ) (Fig. 4B and fig. S9C). In the outDVB, we observe an area expansion ( $\lambda_{A}^{*}>1$ ) up to 2hAPF with a radially decreasing profile. In the DVB, we observe the buildup of a shallower gradient that is transiently paused from 0hAPF to 2hAPF. The contribution to the anisotropic component of the strain ${\underline{\underline{\lambda }}}^{*}$ from changes in cell elongations ${\tilde{\lambda }}_{Q}^{*}$ is small compared to the contribution from cell rearrangements ${\tilde{\lambda }}_{R}^{*}$ (Fig. 4B; fig. S9, A, B, and D; and the “Nematic director pattern on spherical surface” section). While ${\tilde{\lambda }}_{Q}^{*}$ is tangential, following a shallow gradient, ${\tilde{\lambda }}_{R}^{*}$ is radial and increasing with the distance from origin in the outDVB and decreasing in the DVB.

We next use the programmable spring model to test how the observed in-plane cellular behaviors can cause tissue shape changes. We define the DVB and outDVB regions in the model, matching their relative sizes in the wing pouch (Fig. 4C and fig. S10). For simplicity, we use a symmetric spherical cap as an initial geometry. For each individual cell behavior and measured time point (wL3, 0hAPF, 2hAPF, and 4hAPF), we use the in-plane strain ${\underline{\underline{\lambda }}}^{*}$ that we infer from each observed class of cell behaviors as examples of spontaneous strain $\underline{\underline{\lambda }}$. We use these to program the spring lengths in the model. For each insertion of spontaneous strain (model time points: initial, t1, t2, and final, corresponding to the experimental time points), we relax the spring network quasi-statically to a force balanced state (the “Mechanics of the programmable spring lattice” and the “Nematic director pattern on spherical surface” sections). As the effective bending modulus of the wing disc is experimentally inaccessible, we fit the thickness of the model in an example scenario where all observed cell behaviors are input as spontaneous strains and use the same thickness thereafter (fig. S11A and the “Tuning thickness” section).

We first consider cell rearrangements as a possible source of spontaneous strain. When we only input the observed pattern of cell rearrangements as spontaneous strain in the model, ${\underline{\underline{\lambda }}}_{R}=\left\{1,{\tilde{\lambda }}_{R}^{*}\right\}$, it alone creates a strong curvature increase, resembling many features of the data but without increasing tissue size (Fig. 4, A and D). Note that using cell rearrangements as spontaneous strain ${\underline{\underline{\lambda }}}_{R}$ also introduces a difference in curvature change between the two directions, across-DVB and along-DVB, at the final stage.

After relaxing the spring network to a force balanced state, stresses due to residual strains remain. The stresses corresponding to these residual strains can drive passive responses in cell behaviors. The residual strains appear as a mismatch of spontaneous strains (input to the model, $\underline{\underline{\lambda }}$) and strains resulting from changes in spring length during relaxation of the network, $\underline{\underline{F}}$ (Fig. 4E and the “Spontaneous strain tensor” section).

When we calculate the residual strains generated by spontaneous strain from rearrangements, we find that the anisotropic component of the residual strain (${\tilde{\lambda }}_{R}^{\text{res}}$) is tangentially oriented (Fig. 4F and fig. S12D). This tangentially oriented strain is similar to the pattern of cell elongation changes (${\tilde{\lambda }}_{Q}^{*}$) [compare Fig. 4B (middle) with Fig. 4F (right)], suggesting that these cell elongation changes are a passive response to spontaneous strain by rearrangements. To test this idea, we next consider cell elongation as possible source of spontaneous strain. When we only input the observed pattern of cell elongation as spontaneous strain in the model, ${\underline{\underline{\lambda }}}_{Q}=\left\{1,{\tilde{\lambda }}_{Q}^{*}\right\}$, we observe that the spring network shape flattens at the center rather than curve, and cell elongations themselves do not lead to any further residual strains (Fig. 4, G to I, and fig. S12C). This result is consistent with cell elongation changes being a passive response to cell rearrangements and not driving tissue shape change during eversion.

Cell rearrangements as spontaneous strains also lead to residual isotropic compression ( $\lambda_{R}^{\text{res}}$ ; Fig. 4F and fig. S12D). This residual could be compensated by spontaneous area change, which is also required by the observation that overall tissue size increases during eversion (Fig. 4, A and B, and fig. S6, A and B).

When we only input the observed pattern of isotropic strain from cell area changes as spontaneous strain in the model, ${\underline{\underline{\lambda }}}_{A}=\left\{\lambda_{A}^{*},1\right\}$, overall size increases with minimal curvature change (Fig. 4J). This result indicates that although cell area changes are an active behavior and lead to overall size increase, they do not substantially contribute to changes in tissue shape. However, there is a transient effect of cell area changes on tissue curvature at time point t2 (note dip in curve at t2 in Fig. 4J). This transient effect of the spontaneous strain from cell area change only arises from the experimentally observed pause in cell area expansion in the DVB at 2hAPF as compared to the outDVB (fig. S9C, compare DVB and outDVB). This curvature difference disappears when the cell area in the DVB expands to match the outDVB at the final time point, 4hAPF (Fig. 4J). Measuring ${{\underline{\underline{\lambda }}}_{}{}_{R}}^{\text{res}}$ , we find that cell area changes themselves create a small residual in the DVB (Fig. 4, K and L, and fig. S12B). The anisotropic part of this residual could also contribute to the observed passive cell elongations.

Using these examples, we next infer the spontaneous strain patterns that drive tissue shape changes and govern cellular behaviors. We have found that both cell rearrangements and cell area changes are active and contribute to spontaneous strain. We therefore conclude that cell elongation is a passive elastic response and does not contribute to spontaneous strain. The total spontaneous strain, therefore, is composed of the anisotropic part of the observed strain due to rearrangements ( $\tilde{\lambda }{}_{R}^{*}$ ; Fig. 4B), and the isotropic part of the observed cell area changes ( $\lambda_{A}^{*}$ ; Fig. 4B) compensated by the isotropic part of the residual strain due to cell rearrangements ( $\lambda_{R}^{\text{res}}$ ; Fig. 4F). When we input this total inferred spontaneous strain in the model, we find that we can account for the curvature and size changes observed in the everting wing pouch from wL3 to 4hAPF (compare Fig. 4M to Fig. 4A; see also movie S3). The patterns of residual strains generated by the model suggest that after eversion (at 4hAPF), cells experience elongation due to shear stress as well as area constriction due to compressive stresses (Fig. 4, N and O, and fig. S12, A, E, and F). We further tested our model for different thicknesses and geometry of the initial state and did not observe any qualitative differences in the resulting curvature changes (figs. S11B and S13).

In summary, the good qualitative agreement between model output (Fig. 4M) and observed wing pouch curvature changes (Fig. 4A) indicates that the in-plane pattern of spontaneous strain by cell behaviors during eversion is sufficient to capture morphogenesis and that we have identified the most relevant active cellular events responsible for pouch morphogenesis. Specifically, our data predict that altering cell rearrangements in the pouch should have a profound consequence for tissue shape change. We next test this prediction with a genetic perturbation.

### Reduction of active cell rearrangements with MyoVI knockdown disrupts morphogenesis

Previous work in the wing disc pouch of earlier larval stages showed that cell rearrangements drive cell shape patterning (*31*). This work suggested that patterns of active cell rearrangements self-organize via mechanosensitive feedback mediated by Myosin VI (MyoVI). We therefore next investigate whether MyoVI knockdown in the wing pouch (fig. S14A) alters cell rearrangements during eversion and leads to a tissue shape phenotype.

**Fig. 5.**
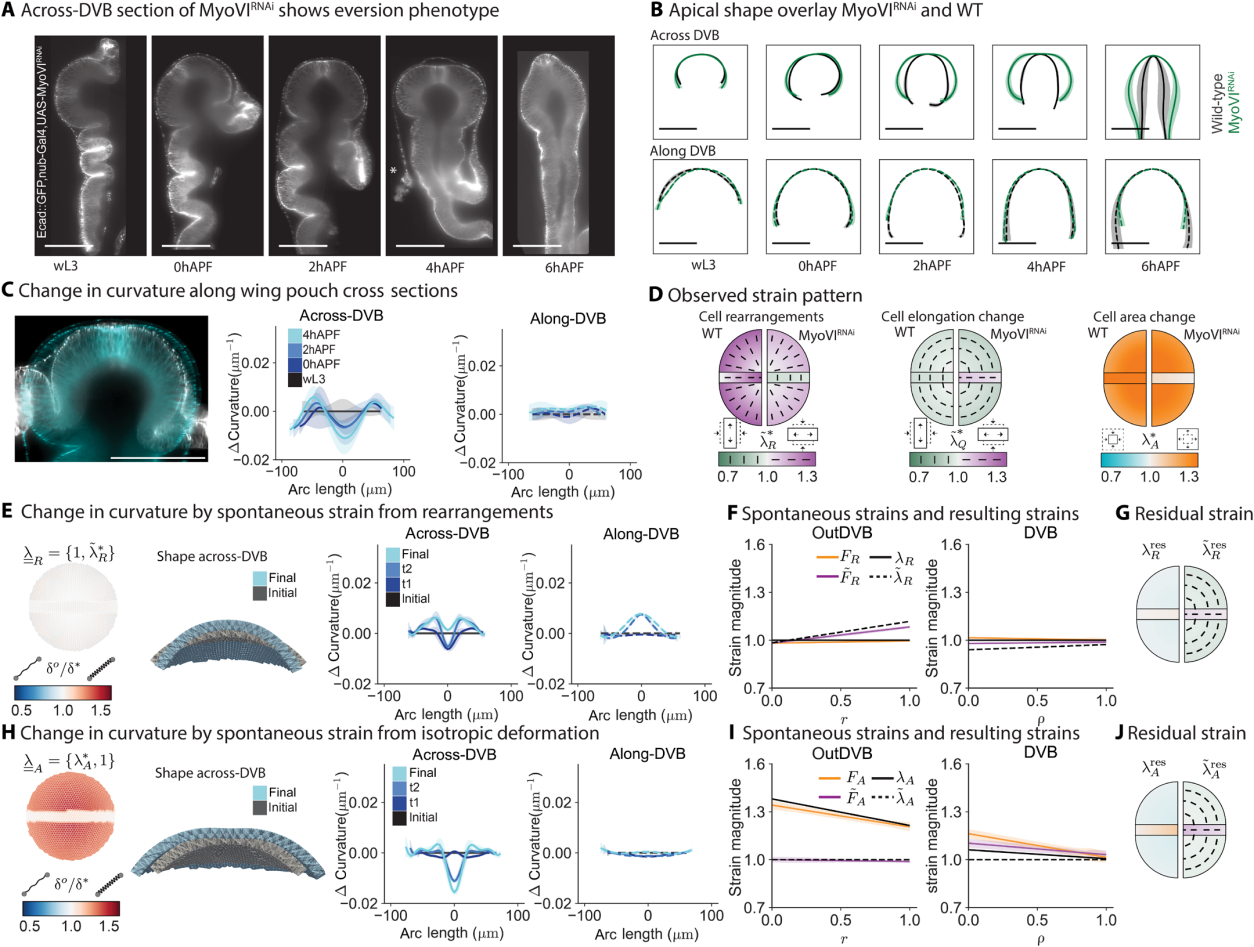
MyoVI^RNAi^ alters active cell behaviors, disrupting morphogenesis. (**A**) Representative across-DVB cross sections of MyoVI^RNAi^ phenotype during eversion. (**B**) Comparison of apical shape between MyoVI^RNAi^ and control. Scale bars, 100 μm. (**C**) Overlay of a wL3 and 4hAPF MyoVI^RNAi^ wing pouch in across-DVB cross section (left) and plots of the average change in tissue curvature in the topologically tracked region for across-DVB (middle) and along-DVB (right) directions. (**D**) Observed strain from cellular behaviors in MyoVI^RNAi^ from wL3 to 4hAPF. Plots are split vertically with the observed strains for WT on the left and MyoVI^RNAi^ on the right. Quarter circles, outDVB; rectangle, DVB. Color represents the strain magnitude; bars indicate orientation of strain for $\tilde{\lambda }{}_{R}^{*}$ and $\tilde{\lambda }{}_{Q}^{*}$ . (**E** and **H**) Observed in-plane behaviors are inserted in the model as spontaneous strains. Springs are colored by new rest lengths normalized by the initial rest lengths (δ*^o^*/δ^*^). Model cross sections show the shape in the across-DVB direction for initial and final time points. t0 matches the shape of WT wL3. t1, t2, and final stages are the model results after a change in spring rest length according to observed strains from 0, 2, and 4hAPF for MyoVI^RNAi^. (**F** and **I**) Input spontaneous strain (λ and $\tilde{\lambda }$ ) at the final eversion time point and comparison with the resulting strain that is achieved after relaxation of the model (*F* and $\tilde{F}$ ). (**G** and **J**) Residual strain from the difference between input and resulting spontaneous strain at the final time point, plotted as in Fig. 4 (F, I, L, and O).

We observe that the MyoVI^RNAi^ wing disc pouch fails to form a flat bilayer after eversion, although its initial shape is similar to that of wild type (WT) (Fig. 5A). This phenotype is best captured in the behavior of curvature in the across-DVB direction (Fig. 5, B and C). Here, the curvature decreases in the center, in contrast to WT, where it increases. In the along-DVB direction, the curvature remains unchanged over time in the MyoVI^RNAi ^knockdown (Fig. 5, B and C, and fig. S14, B to D).

Notably, other features of eversion, such as the opening of the folds and the removal of the peripodial membrane, are unaffected by the MyoVI^RNAi^ knockdown (Fig. 5A; see 4hAPF), indicating that the cause for the altered shape is pouch-intrinsic. This result further supports the idea that tissue shape changes in the wing pouch during eversion are largely independent of other morphogenetic events happening in the wing disc and instead rely on active cell behaviors in the pouch.

Next, we quantify cell behaviors in MyoVI^RNAi^. While initially the gradients in cell areas and elongation are similar to WT (fig. S14, E and F), the inferred strains from individual types of cell behaviors ${\underline{\underline{\lambda }}}^{*}$ differ (Fig. 5D and fig. S15). From work in earlier larval stages, we expect oriented rearrangements to be reduced (*31*). We find that MyoVI^RNAi^ reduces the amount of radial cell rearrangements in the outDVB during eversion (Fig. 5D and fig. S14G). However, in the DVB, rearrangements are of opposite orientation as compared to WT eversion. Notably, we also see a complete lack of cell area expansion in the DVB (Fig. 5D and fig. S14E). The pattern of cell elongations in the outDVB is similar to WT, but in the DVB, it is of perpendicular orientation (Fig. 5D and fig. S14F).

Using the programmable spring model, we test how the reduction of spontaneous strain due to cell rearrangements affects tissue shape changes. When we input only the observed pattern of cell rearrangements in MyoVI^RNAi^ as spontaneous strain in the model, ${\underline{\underline{\lambda }}}_{}{}_{R}=\left\{1,\tilde{\lambda }{}_{R}^{*}\right\}$ , we see only a slight increase in curvature in the final time point in both along- and across-DVB directions (Fig. 5E). Thus, we conclude that the reduction of cell rearrangements in MyoVI^RNAi ^as compared to WT contributes to the abnormal tissue shape changes happening during eversion in MyoVI^RNAi^. We find that the anisotropic component of the residual strain ( $\tilde{\lambda }{}_{R}^{\text{res}}$ ) is small and tangentially oriented in the outDVB and radially in the DVB, similar to the cell elongation pattern (Fig. 5, D and G, and fig. S16D). If we only input observed cell elongation changes as spontaneous strain in the model, ${\underline{\underline{\lambda }}}_{}{}_{Q}=\left\{1,\lambda_{Q}^{*}\right\}$ , we do not recapitulate the observed tissue shape changes (fig. S17). This result suggests that the cell elongation changes in MyoVI^RNAi^ are a passive response to spontaneous strain by cell rearrangements, as in WT.

While the change in spontaneous strain due to rearrangements captures a considerable portion of the difference between the WT and MyoVI^RNAi^ (compare Fig. 5C with Fig. 5E), it fails to recapitulate the finer progression of shape from wL3 to 4hAPF in MyoVI^RNAi^. In particular, the curvature at the final time point of the model calculation is not flattened in the center of the across-DVB direction, and the curvature increases slightly in both directions (Fig. 5, C and E).

Thus, we proceed to input the observed cell area changes as spontaneous strains in the model, ${\underline{\underline{\lambda }}}_{}{}_{A}=\left\{\lambda_{A}^{*},1\right\}$ . We find that they produce shape changes over time similar to those observed during MyoVI^RNAi^ eversion, recapitulating both the decrease in curvature in the center of the across-DVB direction and the lack of curvature change in the along-DVB direction (Fig. 5H and movie S4). We conclude, therefore, that the subtle flattening in the pouch center in MyoVI^RNAi^ during eversion can be explained by the combination of cell area expansion in the outDVB with no area expansion in the DVB. This result highlights that, while cell area changes do not lead to a curvature change in WT, the difference in area expansion between the tissue regions results in the MyoVI^RNAi^ shape. In addition, although we do not observe cell area expansion in the DVB, the area expansion in the outDVB creates residual strains in both regions (Fig. 5, I and J, and fig. S16B). These residual strains have an anisotropic component that, together with the residual strains from cell rearrangements, account for the measured cell elongation patterns in MyoVI^RNAi ^(compare Fig. 5, D, G, and J to fig. S16).

In sum, the results from the MyoVI^RNAi ^perturbation validate the idea that the wing disc pouch deforms like a shape-programmable material. First, by locally perturbing MyoVI, we show that we can alter the normal tissue shape change, although the tissue outside behaves normally, demonstrating that the shape change is tissue autonomous. Second, we show that reducing the active cell rearrangements in the pouch substantially alters the tissue shape outcome, consistent with our theoretical model.

## DISCUSSION

In this work, we show that 3D epithelial tissue morphogenesis in the *Drosophila* wing disc pouch during eversion is based on in-plane spontaneous strains generated by active cellular behaviors. We develop a metric-free, topological method to quantify patterns of cell dynamics on arbitrarily shaped tissue surfaces, as well as a theoretical approach to tissue morphogenesis inspired by shape-programmable materials. These advancements together reveal the mechanics of tissue shape changes during wing disc eversion, showing that active rearrangements and active area expansion govern the 3D tissue shape and size changes.

Using a model with open boundary conditions, we show that programmed cell behaviors on their own are sufficient to generate the observed shape change of the distal wing disc pouch. Therefore, it is not required to have external forces from the surrounding tissue. Although we do not address the behavior of the more proximal tissue here, we observe that it undergoes its own 3D morphogenesis, with the deeply folded regions opening up. How different morphogenetic processes in neighboring tissue regions interact is an interesting avenue for future research.

We hypothesize that the organization of active behaviors during wing pouch eversion arises from patterning during larval growth. First, the prepatterned radial cell area gradient resolves during eversion, giving rise to a gradient of spontaneous strain in the outDVB. Second, the orientation of cell rearrangements follows that of earlier stages, indicating that the mechanosensitive feedback that was revealed in previous work is still active during eversion. Overall, this suggests a developmental mechanism through which mechanical cues at early stages organize cell behavior patterns that later resolve, resulting in a tissue shape change. Such behavior would resemble biochemical prepatterning, in which cell fates are often defined long before differentiation.

Active, patterned rearrangements can robustly give rise to a specific target shape if the tissue is solid on the timescale of morphogenesis. Our work therefore reveals that the everting wing disc behaves as an elastic solid undergoing plastic deformation and demonstrates that the mere presence of rearrangements should not be taken as a sign of a fluid tissue with a vanishing elastic modulus. Many animal tissues with dynamic rearrangements could thus be in the solid regime and therefore be prepatterned toward a target shape. Our work, inspired by shape programmability of complex materials, reveals principles of shape generation that could be quite general. We therefore propose that many other morphogenetic events could and should be considered—and better understood—through the lens of shape programmability.

## MATERIALS AND METHODS

### Experimental model

We performed all experiments with publicly available *Drosophila melanogaster* lines. Flies were maintained at 25°C under 12-hour light/dark cycle and fed with standard food containing cornmeal, yeast extract, soy flour, malt, agar, methyl 4-hydroxybenzoate, sugar beet syrup, and propionic acid. Adult flies were transferred to fresh food two to three times per week. Only males were studied for consistency and due to their smaller size. As WT, we used the F1 offspring of a cross between *w-;ecad::GFP*;; and *w;nub-Gal4,ecad::GFP;;*.

### *D. melanogaster* lines

**Table:** 

Genotype	Construct	Origin
*w-;ecad::GFP;;*	E-cadherin::GFP	Bloomington *Drosophila* Stock Center #60584
*w;nub-Gal4, ecad::GFP;;*	nub-Gal4	Bloomington *Drosophila* Stock Center #86108
*w-;ecad::GFP, UAS-myoVI^RNAi^;;*	MyoVI^RNAi^	Vienna *Drosophila* Resource Center #37534

### Image acquisition and processing

#### Sample preparation

Wing discs of larval stages were dissected in culture medium as previously described (*37*), without surface sterilization or antibiotics. Prepupal stages from 0 to 6hAPF required a slightly different dissection strategy. Prepupae were marked by the time of white pupa formation and collected with a wet brush after the required time interval. Next, pupae were placed on a wet tissue, cleaned with a wet brush to remove residual food, and transferred into glass staining blocks [see (*37*)] filled with dissection medium. To dissect the wing disc, a small cut was performed with fine surgical scissors (2.5 mm, FST 15000-08, Fine Science Tools GmbH) at the posterior end, which created a small hole to release pressure. This allowed for the next cut to be performed at half the anterior-posterior length, separating the anterior and posterior halves. Next, the anterior part of the puparium was first opened at the anterior end by administering a cut just posterior to the spiracles, and then a second cut was performed on the ventral side along the PD axis. The pupal case was then held open with one set of forceps, and a second set of forceps in the other hand was used to remove the wing disc. To dissect wing discs from 0hAPF, the pupa is still soft enough to be turned inside-out after the cut that separates anterior and posterior halves, similar to larval stages.

#### Imaging

Selective Plane Illumination Microscopy (SPIM) imaging was performed with a Zeiss Lightsheet 1 system {488 laser illumination, PCO edge 4.2M Monochrome sCMOS cameras, and a Plan Apo 20× [1.0 numerical aperture (NA)] water immersion objective for detection}. Wing discs were mounted in capillaries (Zeiss, capillary size, 1; inner diameter, ~0.68 mm) with 1% low melting point agarose (LMA; Serva, CAS 9012-36-6). LMA was prepared by mixing 1:1 of Grace’s insect medium and a 2% LMA stock solution made in water. Wing discs were transferred into mounting medium in U-glass dishes, aspirated into the capillary at room temperature, and imaged immediately after the LMA solidified. The imaging chamber was filled with Grace’s insect medium (measured refractive index = 1.3424). For pupal stages, four imaging angles (dorsal, ventral, and two lateral) with 90° rotation were acquired; for larval stages, three imaging angles (dorsal and ventral in one and two lateral) were acquired. Data from dual illumination were fused on the microscope using a mean fusion.

#### Multiview reconstruction

Multiview reconstruction was based on the BigStitcher plugin in Fiji (*38*, *39*). Images were acquired without fluorescent beads, and multiview reconstruction was done using a semiautomated approach. Individual views were manually prealigned. Thereafter, precise multiview alignment was computed on the basis of bright spots in the data with an affine transformation model using the Iterative Closest Point algorithm. Next, images were oriented to show the apical side in *xy* and lateral in *zy*. Last, images were deconvolved using point spread functions extracted from the bright spots and saved as “.tif” files with a manually specified bounding box.

#### Surface extraction of 3D images for visualization

Surfaces shown in Fig. 1B and movie S1 were extracted from 2hAPF and wL3 images. To do so, we first trained a pixel classifier on the strong apical signal of E-cadherin–green fluorescent protein (GFP) of a different image of the same stage with napari-accelerated-pixel-and-object-classification (*40*, *41*). Feature sizes of 1 to 5 pixels were used to predict the foreground on the target image. Next, we used the pyclesperanto library (*42*) to select the largest labels and close gaps in the segmentation with the closing sphere algorithm. For additional gap filling in the 2hAPF time point, we used vedo (*43*) to generate a point cloud and extract the point cloud density. When necessary, we applied some manual pruning of the segmentation in napari (*40*). We repeated this processing on the weak E-cadherin–GFP signal from the lateral membrane and subtracted the apical segmentation from the output. As a result, we achieved a full tissue segmentation that stops just below the apical junction layer. We then extracted the surface using the napari-process-points-and-surfaces (*44*) library and applied smoothing and filling holes. The visualizations were generated using Paraview (*45*). Regions and directions of the cross sections were annotated in Illustrator. Movie S1 was created using Paraview and Fiji (*39*).

### Quantifying curvature of cross sections

Tissue shape analysis was performed on multiangle fused SPIM images. We used Fiji reslicing tools to generate two orthogonal cross sections along the apical-basal direction. Across-DVB is a cross section along the center of the long axis of the wing disc. To find the center, we used the position of the sensory organ precursors and general morphology. The along-DVB cross section follows the DVB and was identified by E-cadherin–GFP signal intensity (fig. S1, A and B). The apical pouch shape was outlined manually along both directions over the pouch region up to the hinge-pouch fold using custom Fiji macros. Subsequent pouch shape analysis was performed in Python. The tissue shape information was extracted from Fiji into Python using the Python “read-roi” package.

The extracted apical shapes were aligned and rotated for each wing disc as follows: First, starting from the left-most point in the curve, we measure the arc length of the curve in the clockwise direction. The arc length of the *i*^th^ point on the curve is given by

$$s\left(i\right)=\sum_{i=2}^{n}\left\|{\underline{x}}_{i}-{\underline{x}}_{i-1}\right\|$$ (1)

where *n* is the number of points in the discrete curve and ${\underline{x}}_{i}=\left(x_{i},y_{i}\right)$ is the position vector of the *i*^th^ point. We keep *s*(*i* = 1) = 0.

Next, we defined the center of the curve at the middle and offset the arc lengths to have *s* = 0 at the center. This leads to negative arc lengths on the left side of the center and positive arc lengths on the right side of the center (figs. S1, C and G, and S6, B and C).

To compute a mean curve from different wing discs of the same developmental stage, we translated and rotated the curves (fig. S1B). We translated each curve by setting their midpoints as the origin (0,0). To rotate the curve, we computed the center of mass of the curve. Then, we defined the new *y* axis as the line that joins the center of mass to the origin. Last, for each curve, we smoothed and interpolated between the discrete points using spline interpolation. We used the *scipy.interpolate.UnivariateSpline* function of scipy (*46*). To smoothen the spline, we defined five knot points, one being the mid-point of the curve, and two others being at three-fourths and half of the length from the mid-point to either side.

Next, we computed the curvature of each curve using the following expression

$$\kappa =\frac{x\prime y\prime \prime -y\prime x\prime \prime }{{\left(x^{\prime 2}+y^{\prime 2}\right)}^{\frac{3}{2}}}$$ (2)

where ′ refers to the derivative with respect to the parameter of the curve, which is arc length in our case.

Last, to compute an average curve, we got the average position vectors at arc lengths starting from a minimum arc length until a maximum arc length in intervals of 5 μm. We did similar averaging for curvature values to get average curvature profiles.

To calculate the change in curvature, we normalized each curve from 0 to 1 and used a linear interpolation with 40 positions to subtract the initial from subsequent curvatures. We then reintroduced the average arc length for each developmental stage for each of the normalized positions.

### Segmentation of the apical junction network

To analyze cell shapes, we used four angles separated by 90° for the segmentation of early pupal stages and a single angle for larval wing discs (fig. S2, A and B). Z-stacks from each imaging angle were denoised if necessary using the N2V algorithm (*47*), and the signal to background ratio was further improved by background subtraction tools in Fiji (*39*). We made 2D projections of the E-cadherin–GFP signal in the disc proper layer as previously described (*48*). This algorithm also outputs a height map image, which encodes the 3D information in the intensity of each pixel. The cells in the wing pouch were segmented using the Fiji plugin Tissue Analyzer and manually corrected (*49*). We chose a bond length cutoff of 2 pixels (∼0.46 μm). The ventral side for 0hAPF was excluded from the analysis, as at this stage, the ventral region is never fully in view from any imaging angle. The number of wing discs per time point and the images for each region are indicated in Table 1. Images were rotated to orient distal down.

**Table 1.** Number of biological replicates for all samples.

Genotype	Stage	Number of wing discs	ROI	Replicates
nub-Gal4, E-cad::GFP (WT)	96hAEL	7	DVB	7
			Dorsal	7
			Ventral	7
	120hAEL	5	DVB	5
			Dorsal	5
			Ventral	5
	wL3	5	DVB	5
			Dorsal	5
			Ventral	5
	0hAPF	7	DVB	7
			Dorsal	7
	2hAPF	5	DVB	5
			Dorsal	5
			Ventral	5
	4hAPF	7	DVB	7
			Dorsal	7
			Ventral	7
	6hAPF	6	DVB	6
			Dorsal	4
			Ventral	4
nub-Gal4, E-cad::GFP, UAS-MyoVI^RNAi^	wL3	5	DVB	5
			Dorsal	5
			Ventral	5
	0hAPF	6	DVB	6
			Dorsal	6
	2hAPF	6	DVB	6
			Dorsal	5
			Ventral	6
	4hAPF	5	DVB	5
			Dorsal	5
			Ventral	5
	6hAPF	7	DVB	7
			Dorsal	6
			Ventral	6

Height map images were rotated accordingly using ImageMagickTM software (ImageMagick Development Team, 2021). We used Fiji macros included with TissueMiner (*50*) to manually specify ROIs. The DVB was identified on the basis of E-cadherin–GFP signal intensity (*51*) and the dorsal versus ventral pouch by their positions relative to global tissue morphology. For larval stages, the DVB, dorsal, and ventral regions were identified in one image. For images showing lateral views of pupal stages, the DVB was identified, whereas for images showing the outDVB region, the dorsal or ventral region and the cells next to the DVB were labeled. The cells next to the DVB were required as a landmark for topological analysis but were otherwise not analyzed separately. We then ran the TissueMiner workflow to create a relational database.

### Live imaging of wing disc eversion

Mounting and imaging for timelapse recording were performed on a wL3 wing disc as described in (*37*). To image wing eversion, a concentration of 400 nM 20-hydroxyecdysone (stock: 2 mM in ethanol source: Sigma-Aldrich, H5142) in Grace’s insect medium with 5% fetal calf serum was used. Explanted upcrawling (116hAEL) wing discs were mounted in uncoated 35-mm glass-bottom dishes (MatTek No. 1.0) and immobilized using porous filters with spacers made from an acid-free double-sided tape. Imaging was performed at 25°C with a spinning disc confocal microscope: Andor IX 83 inverted stand, motorized *xyz* stage with a Prior ProScan III NanoScanZ z-focus device, a Yokogawa CSU-W1 scanhead with Borealis, and a Pecon cage incubator for temperature control. A 60× silicone objective (60×/1.3 NA U Plan SApo, Silicone immersion, Olympus) was used with 488 laser illumination. A 2 by 2 tile of Z-stacks with 0.5-μm spacing were acquired at 5-min intervals with an Andor iXon Ultra 888 Monochrome EMCCD camera. Apical surface projection and segmentation were performed as in (*48*). To facilitate tracking, the movie was split into 10 frame intervals, cell tracks were manually corrected using the Fiji plugin Tissue Analyzer (*49*), and overlapping frames were used to reassign cell tracks for all time points.

### Comparison of live imaging tracks to topological tracking

For the first time point, *k* was specified as in the static data; thereafter, *k* was inherited on the basis of tracked cell IDs throughout all time points. The tracked origin (*k* = 0) was then used to recalculate the topological distance from the origin (*k_i_*) at every time point.

### Number and distribution of cell divisions

Immunostaining was performed on partially dissected wing discs expressing E-cadherin–GFP as previously described (fig. S5A) (*48*). For 6hAPF, wing discs were fully dissected and mounted as described for the timelapse imaging, and immunofluorescence was performed through the porous filter. We used an antibody specific for phosphorylated histone H3 (PH3; Ser^10^) (6G3) (mouse, Cell Signalling catalog no. 9706) and anti-GFP (rabbit, Thermo Fisher Scientific, catalog no. A-11122) in a 1:1000 dilution. Secondary antibodies were goat anti-mouse Alexa Fluor 647 (Thermo Fisher Scientific, catalog no. A-28181) and goat anti-rabbit Alexa Fluor 488 (Thermo Fisher Scientific, catalog no. A-11008) in a 1:1000 dilution. Imaging was performed with a spinning disc confocal microscope (Nikon Eclipse Ti2+ inverted stand, Yokogawa CSU-W scanhead, 488- and 638-nm laser illumination, Hamamatsu Orca Fusion BT camera, run by NIS-Elements ver. 5.42 software). Images were acquired in 0.5-μm spaced Z-stacks using a Nikon 40× objective (Apo LWD 40×/1.15WI lambdaS 0.15-0.19 DIC N2). Data were processed using background subtraction tools from Fiji (*39*). 2D projections were performed on the GFP channel and applied to the PH3 channel with a custom algorithm that identifies the peripodial membrane (PM) and disc proper [see (*48*)].

We additionally used the live imaging data (see the “Live imaging of wing disc eversion” section) and the TissueMiner database (*50*) to computationally identify dividing cells. We plot mother cells that will divide within the following hour of the movie in fig. S5B. For the spatial analysis of cell divisions (fig. S5C), *k*_DV_ was introduced, which is the shortest topological distance to the DVB. *k*_DV_ was calculated for each time point, based on the tracked DVB cells that were assigned on the first time point. The number of dividing cells was thereafter normalized to the number of cells in the topological bin (*k*_DV_ or *k*). For the orientation of division (fig. S5D), Δ*k_i_*, the difference in *k_i_* between the daughter cells in the first frame (5 min) after the cell division was calculated.

### 3D cellular network

We represent the configuration of the cellular network by positions of the cell vertices, where three or more cell bonds meet, and their topological relations as in TissueMiner (*50*). We extended TissueMiner to the third dimension using the information extracted from height maps, as described in the “Segmentation of the apical junction network” section. The code used to generate the curved surface description from TissueMiner is available at https://gitlab.pks.mpg.de/paijmans/CurvedTM.

#### Measurement of cell area and cell elongation tensor

Each cell α in the 3D network contains *N*^α^ vertices ${\underline{v}}_{i}^{\alpha }$ , defining the network geometry. For every cell, we define a centroid ${\underline{R}}^{\alpha }$ , an area *A*^α^, and a unit normal vector ${\underline{N}}^{\alpha }$ as

$${\underline{R}}^{\alpha }=\frac{1}{N^{\alpha }}\;\sum_{i=1}^{N^{\alpha }}{\underline{\upsilon }}_{i},A^{\alpha }=\frac{1}{2}\sum_{i=1}^{N^{\alpha }}\|{\underline{n}}_{i}^{\alpha }\|,{\underline{\hat{N}}}^{\alpha }=\frac{1}{\left\|\sum_{i=1}^{N^{\alpha }},{\underline{n}}_{i}^{\alpha }\right\|}\;\sum_{i=1}^{N^{\alpha }}{\underline{n}}_{i}^{\alpha }$$ (3)

where ${\underline{n}}_{i}^{\alpha }=\left({\underline{v}}_{i+1}^{\alpha }-{\underline{v}}_{i}^{\alpha }\right)\times \left({\underline{R}}^{\alpha }-{\underline{v}}_{i}^{\alpha }\right)$ is the normal vector on the triangle formed by one edge of the cell and the vector pointing from the cell vertex to the cell centroid. It has a norm equal to twice the area of the triangle.

We then create a subcelluar triangulation by connecting the two consecutive vertices in every cell with its centroid $\left\{{\underline{v}}_{i},{\underline{v}}_{i+1},{\underline{R}}^{\alpha }\right\}$ . This creates a complete triangulation that depends both on the vertex positions and the centroids of the cellular network.

Each triangle is defined by its three vertices $\left\{{\underline{R}}_{0},{\underline{R}}_{1},{\underline{R}}_{2}\right\}$ , which define two triangle vectors ${\underline{E}}_{1}\;\text{and}\;{\underline{E}}_{2}$ and its unit normal vector N^

$${\underline{E}}_{1}={\underline{R}}_{1}-{\underline{R}}_{0},\;{\underline{E}}_{2}={\underline{R}}_{2}-{\underline{R}}_{0},\;\hat{\underline{N}}=\frac{{\underline{E}}_{1}\times {\underline{E}}_{2}}{\left\|{\underline{E}}_{1}\times {\underline{E}}_{2}\right\|}$$ (4)

These vectors also define the local basis on the triangle. Using the triangle vectors, we can define the area of the triangle and the rotation angles θ*_x_* and θ*_y_* that rotate a vector parallel to the *z* axis of the lab reference frame to the vector normal to the plane of the triangle

$$\begin{array}{cc} A=\frac{1}{2}\;\left\|{\underline{E}}_{1}\times {\underline{E}}_{2}\right\|, & \theta_{x}=-\text{arctan}\left(N_{y},N_{z}\right),\;\theta_{y}\\ & =\text{arctan}\left(N_{x},1-N_{x}^{2}\right) \end{array}$$ (5)

Here, arctan(*x*, *y*) is the element-wise arc tangent of *x*/*y*, and *N_i_* is a component of the unit vector normal to the triangle plane.

For each triangle, we define the triangle shape tensor ${\underline{\underline{S}}}^{3d}$ as a tensor that maps a reference equilateral triangle with area *A*_0_ lying in the *xy* plane, defined by the vectors ${\underline{C}}_{i}$ to the current triangle

$${\underline{E}}_{i}={\underline{\underline{S}}}^{3d}{\underline{C}}_{i}$$ (6)

The vectors of the reference equilateral triangle are

$${\underline{C}}_{1}=\left(\begin{array}{c} l\\ 0\\ 0 \end{array}\right),\;{\underline{C}}_{2}=\left(\begin{array}{c} l/2\\ \sqrt{3}/2l\\ 0 \end{array}\right),\;{\underline{C}}_{3}=\left(\begin{array}{c} 0\\ 0\\ 1 \end{array}\right)$$ (7)

where the side length $l=\sqrt{4A_{0}/\sqrt{3}}$ with *A*_0_ = 1.

The triangle shape tensor ${\underline{\underline{S}}}^{3d}$ can be written in terms of a planar state tensor ${\underline{\underline{S}}}^{\text{planar}}$ in the reference frame of the triangle as

$${\underline{\underline{S}}}^{3d}={\underline{\underline{R}}}_{}{}_{x}\left(\theta_{x}\right){\underline{\underline{R}}}_{}{}_{y}\left(\theta_{y}\right){\underline{\underline{S}}}^{\text{planar}}$$ (8)

where ${\underline{\underline{R}}}_{}{}_{x}\left(\theta_{x}\right)$ and ${\underline{\underline{R}}}_{}{}_{y}\left(\theta_{y}\right)$ are rotations around the *x* and *y* axis, respectively. The angles θ*_x_* and θ*_y_* are defined in Eq. 5. The planar triangle state tensor, represented by a 3 × 3 matrix with the *z* components set to 0, can be decomposed as

$${\underline{\underline{S}}}^{\text{planar}}=\sqrt{\frac{A}{A_{0}}}{\underline{\underline{R}}}_{}{}_{z}\left(\phi \right)\text{exp}\left(\left\|{\underline{\underline{Q}}}^{}\right\|\underline{\underline{\gamma }}\right){\underline{\underline{R}}}_{}{}_{z}\left(-\phi \right){\underline{\underline{R}}}_{}{}_{z}\left(\theta_{z}\right)$$ (9)

as in TissueMiner. Here, ${\underline{\underline{\gamma }}}^{}$ is a diagonal matrix with diagonal elements {1, − 1,0}, and ${\underline{\underline{R}}}_{}{}_{z}$ is the rotation matrix around the *z* axis. *A* is the area of the triangle, $∥{\underline{\underline{Q}}}^{}∥$ is the magnitude of the elongation tensor, ϕ is the direction of elongation in the *xy* plane, and θ*_z_* is the rotation angle around the *z* axis relative to the reference unilateral triangle. The 3D elongation tensor ${\underline{\underline{\tilde{Q}}}}^{}$ in the lab reference frame and the elongation tensor in the *xy* plane of the triangle ${\underline{\underline{\tilde{Q}}}}^{\text{planar}}$ are related by

$${\underline{\underline{\tilde{Q}}}}^{}={\underline{\underline{R}}}_{}{}_{x}\left(\theta_{x}\right){\underline{\underline{R}}}_{}{}_{y}\left(\theta_{y}\right){\underline{\underline{\tilde{Q}}}}^{\text{planar}}{\underline{\underline{R}}}_{}{}_{x}\left(-\theta_{y}\right){\underline{\underline{R}}}_{}{}_{y}\left(-\theta_{x}\right)$$ (10)

$${\underline{\underline{\tilde{Q}}}}^{\text{planar}}={\underline{\underline{R}}}_{}{}_{z}\left(\phi \right)\text{exp}\left(\left\|\underline{\underline{\tilde{Q}}}\right\|\underline{\underline{\gamma }}\right){\underline{\underline{R}}}_{}{}_{x}\left(-\phi \right)$$ (11)

The magnitude of elongation is calculated as in (*52*).

$$\left\|\underline{\tilde{\underline{Q}}}\right\|=\text{arcsinh}\left(\frac{\left\|{\underline{\underline{S}}}^{\mathrm{ts}}\right\|}{\sqrt{{\left\|{\underline{\underline{S}}}^{\mathrm{ta}}\right\|}^{2}-{\left\|{\underline{\underline{S}}}^{\mathrm{ts}}\right\|}^{2}}}\right)$$ (12)

where $∥{\underline{\underline{S}}}^{\mathrm{ta}}∥$ and $∥{\underline{\underline{S}}}^{\mathrm{ts}}∥$ are the norms of the trace antisymmetric and traceless symmetric part of the planar triangle state tensor ${\underline{\underline{S}}}^{\text{planar}}$ , respectively. The angle of the elongation tensor is given by

$$\phi =\frac{1}{2}\text{arctan}2\left(B_{\mathrm{xy}},B_{\mathrm{xx}}\right)$$ (13)

where *B_ij_* are the components of the nematic part of the triangle state tensor $\underline{\underline{S}}$, and arctan2(*x*1, *x*2) is the inverse tangent of *x*1/*x*2, where the sign of *x*1 and *x*2 is taken into account. In this way, one can select the branch of the multivalued inverse tangent function that corresponds to the angle defined by the point (*x*1, *x*2) in a plane.

We now define the cell elongation tensor as the area-weighted average of the corresponding triangle elongations

$${\underline{\underline{Q}}}^{\alpha }=\frac{1}{A^{\alpha }}\sum_{t\in \text{cell}}a^{t}{\underline{\underline{Q}}}^{t}$$ (14)

where *A*^α^ is the area of the cell, *a^t^* is the area of a triangle that overlaps with the cell, and ${\underline{\underline{Q}}}^{t}$ is the elongation tensor of that triangle.

To calculate the radial component of the cell elongation tensor relative to the origin in cell α, we first define the radial direction. To this end, we use a 3D vector r connecting the origin to the cell centroid, and we project its direction $\underline{r}=\underline{r}/∥\underline{r}∥$ into the tangent plane of the cell, which defines the in-plane radial direction ${\underline{r}}_{\text{tangent}}$ . The tangent plane of the cell is defined by its normal vector $\underline{N}$ defined in Eq. 3 .We calculate the radial components of the cell elongation tensor as

$$Q_{\mathrm{rr}}\equiv {\hat{\underline{r}}}_{\text{tangent}}\cdot {\underline{\underline{Q}}}^{}\cdot {\hat{\underline{r}}}_{\text{tangent}}$$ (15)

relative to the origin.

In the DVB, multiple cells form the origin. To calculate *Q*_ρρ_, the vector $\underline{\rho }$ connects the cell centroid to the averaged position of the topologically nearest cells of *k* = 0. We project its direction $\underline{\rho }=\underline{\rho }/∥\underline{\rho }∥$ into the tangent plane of the cell α, which defines the in-plane direction ${\underline{\rho }}_{\text{tangent}}$ from DVB origin. We calculate the components of the cell elongation tensor as

$$Q_{\rho \rho }\equiv {\hat{\underline{\rho }}}_{\text{tangent}}\cdot {\underline{\underline{Q}}}^{}\cdot {\hat{\underline{\rho }}}_{\text{tangent}}$$ (16)

### Topological distance coordinate system

To calculate topological distances between any two cells, we determine the topological network using the python-igraph library (*53*).

In each of the tissue regions, we define separate origins:

1) outDVB region: To define the origin of the outDVB regions, we first determine the pouch margin cells as cells that live on the outermost row of the segmentation mask and do not overlap with the DVB ROI. Then, for each cell in the region, we calculate the shortest topological distance to the margin cells. This identifies the set of maximally distant cells that have the maximal shortest topological distance to the margin [fig. S3, A (a) and B (a)]. The origin is then defined as the cell that is neighboring the DVB and is at the shortest metric distance to the averaged position of maximally distant cells. At larval stages, both dorsal and ventral sides of the outDVB region are visible, and an origin cell is defined on both sides [fig. S3, A (c) and B (b)].

2) DVB region: We define the origin to consist of a line of cells transversing the DVB. At larval stages, the origin cells are defined as those cells within ${\sqrt{A_{\text{cell}}/\pi }}^{}*1.2$ distance to a straight line connecting the dorsal and ventral center cells (fig. S3A, b and c). For pupal stages, the origin cells for the DVB are defined as the first row of cells next to the margin of the segmentation mask on the distal side (fig. S3C).

The so-identified origin cells serve as the origin for the topological distance (*k*) for each cell in the tissue. In this way, *k* follows the radial direction along the surface for the outDVB and the path along the the DVB for the DVB.

### 3D visualization of cell properties

We visualize cellular properties and cell elongation tensors on the 3D segmentation mask using Paraview (*45*). To plot a rank 2 tensor, like the cell elongation tensor, we take the largest eigenvalue of ${\underline{\underline{Q}}}^{\alpha }$ as the norm of elongation and the corresponding eigenvector as the direction of elongation that we can plot to the surface. Note that for cells/patches that are reasonably flat, the eigenvector with the eigenvalue closest to zero is (almost) parallel to the normal vector on the patch.

### Spatial analysis of cell properties

We acquired data for five to seven wing discs of each developmental stage (see Table 1). Images that were not of segment-able quality were excluded from the analysis. We average cell properties by *k* between dorsal and ventral for the outDVB and between images from both sides of the DVB. We used a cell area–weighted average for elongation. The 95% confidence interval and the statistic mean for each developmental stage are calculated via bootstrap resampling with 10,000 repeats.

### Mechanics of the programmable spring lattice

We used a programmable spring lattice in the shape of a symmetric spherical cap to model the wing disc pouch, which is an epithelial monolayer.

#### Approximating the wing disc pouch as a spherical cap

We calculated the average radius of curvature of the apical side of the wing disc pouch at wL3 stage in the topologically tracked region as *R* = 77.66 μm. The angular size of the spherical cap, denoted by θ*_M_*, is given by

$$\theta_{M}=\frac{1}{2}\left(\frac{w_{\text{DV}}+2w_{\text{ODV}}}{R}\right)$$ (17)

where *w*_DV_ is the width of the DVB and *w*_ODV_ is the average in-surface distance from the DVB to the periphery of the outDVB region (fig. S10A). We calculated *w*_DV_ = 15 μm and *w*_ODV_ = 59.77 μm. Using these calculated dimensions, we determined θ*_M_* = 49.63^∘^.

#### Generating the lattice

We first generated a triangular lattice in the shape of a hollow sphere, keeping the radius of curvature *R* calculated above. This lattice was obtained using the function *meshzoo*. *icosa*_*sphere* available in the Python package Meshzoo (www.github.com/meshpro/meshzoo). In this function, we set the argument refine_factor = 30, which leads to edges of length 3.11 ± 0.18 μm. This edge length was found to be small enough to prevent computational errors in the simulations of this study. We then cropped the spherical lattice to obtain a spherical cap of angular size θ*_M_* (calculated above; fig. S10B). Next, we placed a second layer at the bottom of this lattice at a separation of *h*. This new layer is identical to the original lattice in terms of the topology of the lattice network but is rescaled to have a radius of curvature of *R-h*. We connected the two layers with programmable springs using the topology shown in the inset of fig. S10C. The lattice obtained this way represents an elastic surface of thickness *h*, which can be changed to tune the bending rigidity of the model. Vertices typically have 13 neighbors (6 on their own layer and 7 on the other layer). However, six to eight vertices of about 3220 vertices in the whole network form point defects. These vertices have 11 neighbors.

To remove any possible effects coming from the lattice structure (angle of edges or degree of connectivity), we performed simulations for each condition by taking spherical caps from 50 different regions of the sphere and averaging the result. We saw only very small variability in the final shape, quantified by the SD of the curvature change profiles in our model results. Thus, we conclude that the lattice structure does not affect our results.

#### Elastic energy of model

The edges of the lattice act as overdamped elastic springs with rest lengths equal to their initial lengths. Hence, the model is stress free at *T* = 0

$$\delta_{I}^{a}=\left\|\Delta {\underline{X}}^{a}\right\|$$ (18)

where *a* denotes a single spring; $\Delta {\underline{X}}^{a}$ denotes the spring vector given by ${\underline{X}}^{\beta }-{\underline{X}}^{\alpha }$ , where α and β are the vertices at the two ends of spring *a*, and ${\underline{X}}^{\alpha }$ denotes the position vector of vertex α. During a consequent time step *T*, the rest length of spring *a* ( $\delta_{T}^{a}$ ) can differ from its current length δ. The elastic energy of this state for the whole lattice is given by

$$W=\frac{1}{2}\sum_{a}\;k{\left(\delta^{a}-\delta_{T}^{a}\right)}^{2}$$ (19)

where the sum is over all springs of the network and *k* represents the spring constant. At each computational time step *T*, the model tries to find a preferred configuration by minimizing *W*; hence, *T* acts as a “quasi-static time step.” To minimize the energy of the model at a given *T*, we used overdamped dynamics with smaller time steps τ, which restart for each new quasi-static time step *T*.

$$\begin{array}{ll} \frac{d{\underline{x}}^{\alpha }}{\mathrm{d\tau}} & =-\frac{1}{\gamma }\frac{\partial W}{\partial {\underline{x}}^{\alpha }}\\ & =-\frac{k}{\gamma }\sum_{a}\left(\delta^{a}-\delta_{T}^{a}\right){\hat{\underline{\delta }}}^{a} \end{array}$$ (20)

Here, γ represents the friction coefficient. ${\underline{x}}^{a}$ corresponds to the current position of the vertex α. δ*^a^* is the length of the springs connected to vertex α. ${\underline{\hat{\delta }}}^{a}=\left({\underline{x}}^{\alpha }-{\underline{X}}^{\beta }\right)/\delta^{a}=\left(\Delta {\underline{x}}^{a}\right)/\delta^{a}$ represents the unit vector along the spring *a* that connects vertices α and β.

We relaxed the model at each quasi-static time step *T* to achieve force balance by updating the positions of the particles using

$${\underline{x}}^{\alpha }\left(\tau +d\tau \right)={\underline{x}}^{\alpha }\left(\tau \right)-d\tau \frac{k}{\gamma }\sum_{a}\left(\delta^{a}-\delta_{T}^{a}\right){\underline{\hat{\delta }}}^{a}$$ (21)

where $d\tau \frac{k}{\gamma }$ was set to 0.01 (ensuring no numerical artifacts).

The particles were moved until the average movement of the particles $\langle ∥{\underline{x}}^{a}\left(\tau +d\tau \right)-{\underline{X}}^{\alpha }\left(\tau \right)∥\rangle /R$ reduced to 10^−9^, where *R* is the radius of curvature of the outer surface of the spherical cap in the initial stress-free state.

### Spontaneous strain tensor

Tissue shape change during development is modeled in this work as the appearance of spontaneous strains, a change in the ground state of local length scales. This notion can be captured with a spontaneous strain tensor field, $\underline{\underline{\lambda }}\left(\underline{X}\right)$, a rank 2 tensor. Each component corresponds to the multiplicative factor by which the rest lengths of the material changes in a particular direction. In some general coordinate system, we can write $\underline{\underline{\lambda }}$ as

$$\underline{\underline{\lambda }}=\left(\begin{array}{ccc} \lambda_{11} & \lambda_{12} & \lambda_{13}\\ \lambda_{21} & \lambda_{22} & \lambda_{23}\\ \lambda_{31} & \lambda_{32} & \lambda_{33} \end{array}\right)$$ (22)

We chose the coordinate system so that it aligns with our desired deformation pattern. In this case, $\underline{\underline{\lambda }}\left(\underline{X}\right)$ is in a diagonal representation

$$\underline{\underline{\lambda }}=\lambda_{11}\left({\underline{e}}_{1}⊗{\underline{e}}_{1}\right)+\lambda_{22}\left({\underline{e}}_{2}⊗{\underline{e}}_{2}\right)+\lambda_{33}\left({\underline{e}}_{3}⊗{\underline{e}}_{3}\right)$$ (23)

where the basis vectors were chosen such that ${\underline{e}}^{1}\;\text{and}\;{\underline{e}}^{2}$ are the surface tangents, while ${\underline{e}}^{3}$ is the surface normal. In general, we kept λ_33_ = 1, since we do not input any spontaneous strains along the thickness of the model.

The surface components of $\underline{\underline{\lambda }}$ can be further broken down into isotropic and anisotropic components. Isotropic deformation changes the local area of the surface by changing the local lengths equally in all directions. Anisotropic deformation increases the local length in one direction while decreasing the local length in the other direction so as to preserve the local area. Thus, we decompose the deformation as a product of isotropic and anisotropic contributions.

$$\begin{array}{c} \lambda_{11}=\lambda \tilde{\lambda }\\ \lambda_{22}=\lambda {\tilde{\lambda }}^{-1} \end{array}$$ (24)

Then, the spontaneous deformation tensor can be written as

$$\underline{\underline{\lambda }}=\lambda \tilde{\lambda }\left({\underline{e}}_{1}⊗{\underline{e}}_{1}\right)+\lambda {\tilde{\lambda }}^{-1}\left({\underline{e}}_{2}⊗{\underline{e}}_{2}\right)+\left({\underline{e}}_{3}⊗{\underline{e}}_{3}\right)$$ (25)

Last, as $\underline{\underline{\lambda }}$ is a field, each of the components in the above equation generally depends on the location on the surface, $\underline{X}$.

#### *Discretizing* $\underline{\underline{\lambda }}$

As our spring lattice is discrete in nature, we used the following strategy to discretize $\underline{\underline{\lambda }}$. For a single spring, $\underline{\underline{\lambda }}$ is an average of the value of $\underline{\underline{\lambda }}$ on the two ends of the springs.

$${\underline{\underline{\lambda }}}^{a}=\frac{1}{2}\left[\underline{\underline{\lambda }}\left({\underline{X}}^{\alpha }\right)+\underline{\underline{\lambda }}\left({\underline{X}}^{\beta }\right)\right]$$ (26)

where α and β are the two vertices of the spring *a*.

#### Assigning new rest lengths to springs

The initial length of spring *a* connecting vertices α and β is given by

$$\delta_{I}^{a}=\left\|{\underline{X}}^{\alpha }-{\underline{X}}^{\beta }\right\|=\left\|\Delta {\underline{X}}^{a}\right\|$$ (27)

To assign new rest lengths, we used

$$\delta_{F}^{a}=\left\|{\underline{\underline{\lambda }}}^{a}.\Delta {\underline{X}}^{a}\right\|$$ (28)

Note that we assigned a new rest length to any spring *a* based on the positions of its vertices ( ${\underline{X}}^{\alpha }$ and ${\underline{X}}^{\beta }$ ), independent of the layer in which these vertices lie (top and bottom). Accordingly, top and bottom springs at any position have their rest lengths updated by the same amount.

#### Implementing shape change over time

We increased the spontaneous strain slowly to model the slow build up of stresses due to cell behaviors. Hence, we first calculated the target rest length of springs ( $\delta_{F}^{a}$ ). At each time step, we assigned a rest length $\delta_{T}^{a}$ and minimized the energy of the model. We increased $\delta_{T}^{a}$ in a simple linear manner from $\delta_{I}^{a}$ to $\delta_{F}^{a}$

$$\delta_{T}^{a}=\delta_{I}^{a}+\left(\delta_{F}^{a}-\delta_{I}^{a}\right)\frac{T}{T_{F}}$$ (29)

where *T_F_* is the number of quasi-static time steps in which the whole simulation takes place. Note that within each time step, the lattice was brought to a force balance state. The simulations were performed for different choices of *T_F_* (1, 2, and 5), but we found that the differences in output shapes were undetectable. Still, *T_F_* = 5 was chosen to simulate the slow appearance of spontaneous strains.

#### Measuring resulting strains in model

In our spring model, displacements were defined by positions of vertices, and we defined the deformation gradient tensor ${\underline{\underline{F}}}^{\alpha }$ at each vertex α of the network.

For each spring *a* emerging from the vertex α, the deformation gradient tensor should satisfy

$${\underline{x}}^{a}={\underline{\underline{F}}}^{\alpha }{\underline{X}}^{a}$$ (30)

However, ${\underline{\underline{F}}}^{\alpha }$ contains 9 df, while there are 13 springs for each vertex and therefore 13 independent equations to be satisfied. Note that six to eight vertices of about 3220 vertices in the whole network form point defects and thus have 11 springs. Therefore, we defined ${\underline{\underline{F}}}^{\alpha }$ as the tensor that best satisfies conditions in Eq. 30 by minimizing the sum of residuals squared

$$S=\sum_{a}{\left\|{\underline{\underline{F}}}^{\alpha }{\underline{X}}^{a}-{\underline{x}}^{a}\right\|}^{2}$$ (31)

This is an ordinary least squares (OLS) problem split into three independent basis vectors. We solved this OLS using the Numpy method *numpy*. *linalg*. *lstsq* in Cartesian coordinates (*54*). We then expressed ${\underline{\underline{F}}}^{}$ in the coordinate system corresponding to vertex α in the model explained above. From this, we calculated the isotropic (*F*) and anisotropic ( $\tilde{F}$ ) components using

$$F=\sqrt{F_{\mathrm{rr}}F_{\phi \phi }}$$ (32)

$$\tilde{F}=\sqrt{F_{\mathrm{rr}}/F_{\phi \phi }}$$ (33)

Last, we computed ${\underline{\underline{\lambda }}}^{\text{res}}$ as

$${\underline{\underline{\lambda }}}^{\text{res}}={\underline{\underline{F}}}^{}{\underline{\underline{\lambda }}}^{-1}$$ (34)

The isotropic (λ^res^) and anisotropic components ( $\tilde{\lambda }{}^{\text{res}}$ ) of ${\underline{\underline{\lambda }}}^{\text{res}}$ are calculated in the same way as for ${\underline{\underline{F}}}^{}$.

### Nematic director pattern on spherical surface

In the initial state of the model, we specified a coordinate system on the spherical surface in different regions (outDVB and DVB). These coordinate systems were chosen such that the observed nematic patterns of spontaneous strains ( $\tilde{\lambda }$ ) aligned with the major axes of the chosen coordinate systems.

We first defineed the origins in our model similar to the origins defined in the data (Fig. 3C and fig. S10C). To do so, we first measured θ_DV_. The coordinates of *O*_D_ and *O*_V_ are then given by [±*R* sin (θ_DV_/2),0, *R* cos (θ_DV_/2)] in the Cartesian coordinate system. The center for the DVB region is given by the line *O*_DV_ which joins *O*_D_ and *O*_V_.

In the outDVB region, we had a coordinate system in which the basis vectors are given by ${\underline{e}}_{r},{\underline{e}}_{\phi },{\underline{e}}_{h}$ (fig. S10, C and D). ${\underline{e}}_{r}$ is simply the normal vector on the spherical surface. To calculate ${\underline{e}}_{r}$ at a point, we drew a vector from the origin in this region (*O*_D_ or *O*_V_) to the point. We then took a projection of this vector onto the tangent plane of the surface and normalized it to give us a unit vector. In this way, we calculated ${\underline{e}}_{r}$ as a surface tangent vector emanating radially outward from the origins of the outDVB regions. ${\underline{e}}_{\phi }$ is then the direction perpendicular to ${\underline{e}}_{r}$ and ${\underline{e}}_{h}$ . For each point in the outDVB region, we calculated the geodesic distance between the point and the center point of its region. We then normalized this distance by the maximum geodesic distance from the center calculated in this region. This gave us a scalar coordinate *r* which varies from 0 to 1.

In the DVB region, the basis vectors are given by $\left({\underline{e}}_{\rho },{\underline{e}}_{w},{\underline{e}}_{h}\right)$ (fig. S10, C and D). ${\underline{e}}_{h}$ is simply the normal vector on the spherical surface. To calculate ${\underline{e}}_{\rho }$ at a point, we drew a vector from the nearest point on *O*_DV_ to the point. We then took a projection of this vector onto the tangent plane of the surface and normalized it to give us a unit vector. In this way, we calculated ${\underline{e}}_{\rho }$ as a surface tangent vector emanating outward from the center line of the DVB region as well as parallel to the DVB. ${\underline{e}}_{w}$ is perpendicular to ${\underline{e}}_{\rho }$ and ${\underline{e}}_{h}$ . For each point in the DVB region, we calculated the shortest distance between the point and the center line of the DVB region. We then normalized this distance by the maximum distance from the center line in the DVB region. This gave us a scalar coordinate ρ.

For the simple examples presented in Fig 2C (except Fig 2C, c), θ_DV_ was set to be 0 to have a simple radial coordinate system. For Fig 2C (c), θ_DV_ > θ_M_.

### Extracting the strain pattern from segmented images

To quantify the strain due to different cell behaviors along the basis vectors of the chosen coordinate system, we compared cells within topologically tracked bins between two different developmental stages.

#### Tracking location between developmental stages

We leveraged the topological distance coordinate system to track locations between discs. Each topological ring *k* is given a value *N* which denotes the cumulative number of cells from the topological origin defined in each region (*O*_D_, *O*_V_, and *O*_DV_). We used *N* to track the location in our static images of different discs at different developmental stages.

#### Observed strain due to cell area change

Cell area scales with the square of the distance between cell vertices. Thus, the factor by which the local lengths change in all directions is given by

$$\lambda_{A}^{*}\left(N\right)=\sqrt{\frac{A\left(N,t+\Delta t\right)}{A\left(N,t\right)}}$$ (35)

Here, *t* corresponds to an initial developmental stage, and *t* + Δ*t* corresponds to a later developmental stage. *A* refers to the average cell area evaluated at *N*.

#### Observed strain due to cell elongation change

Each cell is given a cell elongation tensor ${\underline{\underline{Q}}}^{}$ that is the average of further subdivisions of the cell polygon into triangles (the “3D cellular network” section). Each triangle can be circumscribed by an ellipse, the centroid of which coincides with the centroid of the triangle. According to (*55*), the length of the long axis of the ellipse is given by $l=r_{o}\text{exp}\left(∥{\underline{\underline{Q}}}^{}∥\right)$ , where *r*_o_ is the radius of a reference equilateral triangle. The length of the short axis of the ellipse is given by $s=r_{o}\text{exp}\left(-∥{\underline{\underline{Q}}}^{}∥\right)$ . The axes of the ellipse match with the radial and tangential directions if the off-diagonal components *Q*_*r*ϕ_ or *Q*_ρϕ_ are approximately 0. This was the case for our data as well. The length scale associated with the radial direction is *l* if *Q_rr_* or *Q*_ρρ_ is positive and *s* if *Q_rr_* or *Q*_ρρ_ is negative. Thus, we got a measure of the length scales along the radial direction, which we denote by *L* and is given by

$$L=\text{exp}\left(\sigma \left\|\tilde{{\underline{\underline{Q}}}^{}}\right\|\right)$$ (36)

where σ is the sign of *Q_rr_* or *Q*_ρρ_.

We then averaged *L* within each ring and computed a ratio of the length scales along the radial direction between two developmental stages by computing

$$\tilde{\lambda }{}_{Q}^{*}\left(N\right)=\frac{L\left(N,t+\Delta t\right)}{L\left(N,t\right)}$$ (37)

#### Observed strain due to cell rearrangements

Rearrangements lead to anisotropic deformation of the tissue. In our topological coordinate system, radially oriented rearrangements lead to an increase in the number of rings needed to accommodate some fixed number of cells (fig. S8). Similarly, tangential rearrangements would lead to a decrease in the number of topological rings. Thus, by measuring the change in the number of rings needed to accommodate some fixed number of cells, we can estimate the deformation due to the net effect of radial and tangential rearrangements.

In a tissue region at developmental stage *t*, let us consider a single ring with index *k* and cumulative number of cells *N*. Ring *k* contains Δ*N* cells given by *N*(*k*, *t*) − *N*(*k* − 1, *t*). By construction, the number of rings needed to contain Δ*N* cells at location *N* is given by *n*(*N*, *t*) = 1. For a later developmental stage, *t* + Δ*t*, we estimated *n*(*N*, *t* + Δ*t*) which is the number of rings that contain Δ*N* cells at the location *N*. This is done by taking the difference between *k* values evaluated at *t* + Δ*t* and at locations *N*(*k* − 1, *t*) and *N*(*k*, *t*) (see also fig. S8)

n(N,t+Δt)=k[N(k,t),t+Δt]−k[N(k−1,t),t+Δt] (38)

As *n*(*N*, *t*) and *n*(*N*, *t* + Δ*t*) are measures of the number of topological rings, they represent the radial topological length scales that change due to cell rearrangements. Thus, the strain due to cell rearrangements is quantified by

$$\lambda_{R}^{*}\left(N\right)=\frac{n\left(N,t+\Delta t\right)}{n\left(N,t\right)}$$ (39)

$\lambda_{R}^{*}\left(N\right)>1$ represents radial extension of the tissue due to radially oriented rearrangements, while $\lambda_{R}^{*}\left(N\right)<1$ represents tangential extension.

#### Observed strain due to combination of cell elongation change and cell rearrangements

The combined strain due to cell elongation change and cell rearrangements is given by

$$\tilde{\lambda }{}^{*}\left(N\right)=\tilde{\lambda }{}_{Q}^{*}\left(N\right)\tilde{\lambda }{}_{R}^{*}\left(N\right)$$ (40)

#### Mapping locations between wing disc images and model

In the model, we have a dimensionless scalar coordinate in the outDVB and DVB regions varying from 0 to 1. In the data as well, we prescribed a scalar coordinate to each topological bin. To do so, we calculated the path length in micrometers of the shortest path along cell centers from each cell to the origin and averaged this path length for each topological bin. For each bin, we normalized this path length by the average path length of the outermost topological bin in the corresponding region (DVB or outDVB). We called this normalized path length *r* for the outDVB region and ρ for the DVB region. Because of our normalization, *r* and ρ run from 0 to 1, similar to the model.

Thus, we were able to map any topological ring (identified by *k* and *N*) to a scalar coordinate *r* in outDVB or ρ in DVB. In the “Nematic director pattern on spherical surface” section, we explain the mapping between *r* and ρ to the Cartesian coordinates of the vertices in the model given by ${\underline{X}}^{\beta }\alpha$, where α is a vertex. Using this mapping, any strain component, for example $\tilde{\lambda }{}_{R}^{*}\left({\underline{X}}^{\alpha }\right)$ , on the model vertex α can be evaluated from a corresponding $\tilde{\lambda }{}_{R}^{*}\left(N\right)$.

### Quantifying curvature of model cross sections

To quantify the curvature of the model output, we first isolate the top layer of the lattice. Then, we take the along-DVB cross section (*xz* plane) and the across-DVB cross section (*yz* plane). To take the cross section, we record the points of intersection of the in-surface springs with the respective plane of the cross section. From this, we get a discrete set of points that are ordered along their horizontal position to get a counterclockwise curve. These curve data are now similar to the data we obtain from segmented images. Hence, we apply the exact same procedure described above to quantify the curvature of the model output.

### Tuning thickness

We tuned the thickness of the model to change the bending modulus. We first performed simulations by inputting all cell behaviors (cell area changes, cell elongation changes, and cell rearrangements) combined as spontaneous strains. We performed this simulation for different thicknesses *h/R* = 0.05, 0.1, and 0.15, where *h* is the thickness of the model and *R* is the radius of curvature of the top surface of the initial state of the model (fig. S11A). We found that *h*/*R* = 0.1 gives us the best matching of the curvature change profiles with the wing disc pouch. Fixing *h*/*R* = 0.1, we performed further analysis to infer the spontaneous strains in the wing disc pouch. Inputting these inferred spontaneous strains, we again performed simulations for different thicknesses. We found that *h*/*R* = 0.1 still matches the wing disc pouch curvature change values best (fig. S11B).

### Testing the model with more realistic initial geometry

In our model, we used a spherically symmetric initial geometry, whereas the pouch is slightly asymmetric in the along-DVB and across-DVB cross sections (fig. S6C). To determine whether the anisotropy of the initial shape plays a crucial role in the shape evolution, we extracted meshes of individual apical surfaces from the cell segmentations of five wing disc pouch tissues at wL3 stage. The cell segmentation was manually categorized into a DVB region and two outDVB regions, and the origins *O*_D_, *O*_V_, and *O*_DV_ were previously computed (see the “Topological distance coordinate system” section). For each cell center, the path length to the origin of their respective region and the cell normals were calculated. The scalar coordinates ρ and *r* were calculated by normalizing the path lengths of the cell centers by the maximum path length in their respective regions. Knowing the origins and the cell normals ( ${\underline{e}}_{h}$ ), the basis vectors for each cell center were computed in a similar manner as explained in the “Nematic director pattern on spherical surface” section. Each cell center is a vertex in the mesh that is created this way. To add thickness to this mesh, for each vertex, we added a new vertex that is displaced by a length of *h* = 0.1*R* along $-{\underline{e}}_{h}$ . Here, *R* is the average radius of curvature of the pouch. These new vertices form the second layer of the mesh and are connected to the first layer of the mesh in the same way as for the spherical mesh. Using these meshes as the initial state of the model, we observe more noisy but qualitatively and quantitatively similar results as in the symmetric case (fig. S13).
